# In Situ Super-Hindrance-Triggered Multilayer Cracks for Random Lasing in π-Functional Nanopolymer Films

**DOI:** 10.34133/research.0027

**Published:** 2023-01-16

**Authors:** Dongqing Lin, Yang Li, He Zhang, Shuai Zhang, Yuezheng Gao, Tianrui Zhai, Shu Hu, Chuanxiang Sheng, Heng Guo, Chunxiang Xu, Ying Wei, Shifeng Li, Yelong Han, Quanyou Feng, Shasha Wang, Linghai Xie, Wei Huang

**Affiliations:** ^1^ Centre for Molecular Systems and Organic Devices (CMSOD), State Key Laboratory of Organic Electronics and Information Displays & Institute of Advanced Materials (IAM), Nanjing University of Posts and Telecommunications, 9 Wenyuan Road, Nanjing 210023, China.; ^2^ College of Physics and Optoelectronics, Faculty of Science, Beijing University of Technology, Beijing 100124, China.; ^3^ School of Electronic and Optical Engineering, Nanjing University of Science and Technology, Nanjing 210094, China.; ^4^ State Key Laboratory of Bioelectronics, School of Biological Sciences & Medical Engineering, Southeast University, Nanjing 210096, China.; ^5^ College of Engineering and Applied Science, Nanjing University, Nanjing, 210023, China.; ^6^ Frontiers Science Center for Flexible Electronics (FSCFE), MIIT Key Laboratory of Flexible Electronics (KLoFE), Northwestern Polytechnical University, Xi'an 710072, China.

## Abstract

In situ self-assembly of semiconducting emitters into multilayer cracks is a significant solution-processing method to fabricate organic high-*Q* lasers. However, it is still difficult to realize from conventional conjugated polymers. Herein, we create the molecular super-hindrance-etching technology, based on the π-functional nanopolymer PG-Cz, to modulate multilayer cracks applied in organic single-component random lasers. Massive interface cracks are formed by promoting interchain disentanglement with the super-steric hindrance effect of π-interrupted main chains, and multilayer morphologies with photonic-crystal-like ordering are also generated simultaneously during the drop-casting method. Meanwhile, the enhancement of quantum yields on micrometer-thick films (*Φ* = 40% to 50%) ensures high-efficient and ultrastable deep-blue emission. Furthermore, a deep-blue random lasing is achieved with narrow linewidths ~0.08 nm and high-quality factors *Q* ≈ 5,500 to 6,200. These findings will offer promising pathways of organic π-nanopolymers for the simplification of solution processes applied in lasing devices and wearable photonics.

## Introduction

Organic solid-state lasers are fascinating for full-color display arrays [1], flexible photonics [2], intelligent sensors [3], and optical on-chips [4,5], based on π-functional gain media [6] with large optical cross sections [7], well-tuned refractive indices [8], well-tailored luminescence wavelengths, and large-scale solution processability [9]. Generally, lasing performances from organic gain media are achieved by additionally introducing photonic crystals (PhCs) like distribution Bragg reflection (DBR) and distribution feedback Bragg cavities [10], which require multistep and expensive fabrication methods including lithography, etching, and imprint [11,12]. To simplify the fabrication of lasing systems, random lasers consisting of gain medium and nanoparticles have emerged [13*–*15], where metal or dielectric nanoparticles provide plasmonic resonance or multiple scattering for optical feedback. Compared with random lasers from colloidal semiconductor nanostructures [16,17], organic and polymer semiconductors can achieve simple-component random lasers [18*–*20] without extra microcavities or nanoparticles. Through the modulation of self-assembly behaviors, organic and polymer semiconductors naturally form Fabry–Perot (from nanowire-like architectures [20]) or whispering-gallery-mode resonators (from micro/nanosheets [21] or microspheres [22,23]). These well-ordered microstructures can avoid serious optical losses from the nanoparticle-induced random scattering [24] (additionally with ohmic losses from metal nanoparticles [25]), which is favorable to lasing performances with low thresholds and high-quality factors (*Q*). In particular, organic high-*Q* random lasers are a crucial step toward electrically pumped lasing [26], together with attractive functions [3,9] involving sensing, spectroscopy source, displays, and communication applications. However, most organic single-component random lasers suffer from considerable difficulties in achieving quasi-continuous-wave lasing with ultranarrow linewidths and high-*Q* performances. Thus, it is essential to tailor newly self-assembled architectures based on innovating morphology-directed molecular design.

Introducing interface cracks is an intriguing strategy to manipulate optical scattering and diffraction for the improvement of lasing performances [27]. Although interface cracks usually decrease mechanical toughness and carrier mobility [28], they can intrinsically possess large refractive index contrast (∆*n* > 0.5 to 0.8) to act as natural cavities [29]. So far, a variety of top-down multicomponent processes, including electrochemical anodization [30], ion-beam etching [31], and lift-off-based patterning [32], have been employed to induce interface cracks in a sophisticated, multistep, and expensive manner. Inspired by the reverse mode of self-healing polymers with strong supramolecular association [33], we envisage that interface cracks can be spontaneously formed by inducing disentanglement behaviors during the self-assembly process of π-functional polymers. Toward this, we utilize the super-hindrance effect of gridarene-based π-nanopolymers to deliberately induce massive interface cracks, which are also integrated with DBR-like multilayer architectures during the drop-casting process.

**Fig. 1.**
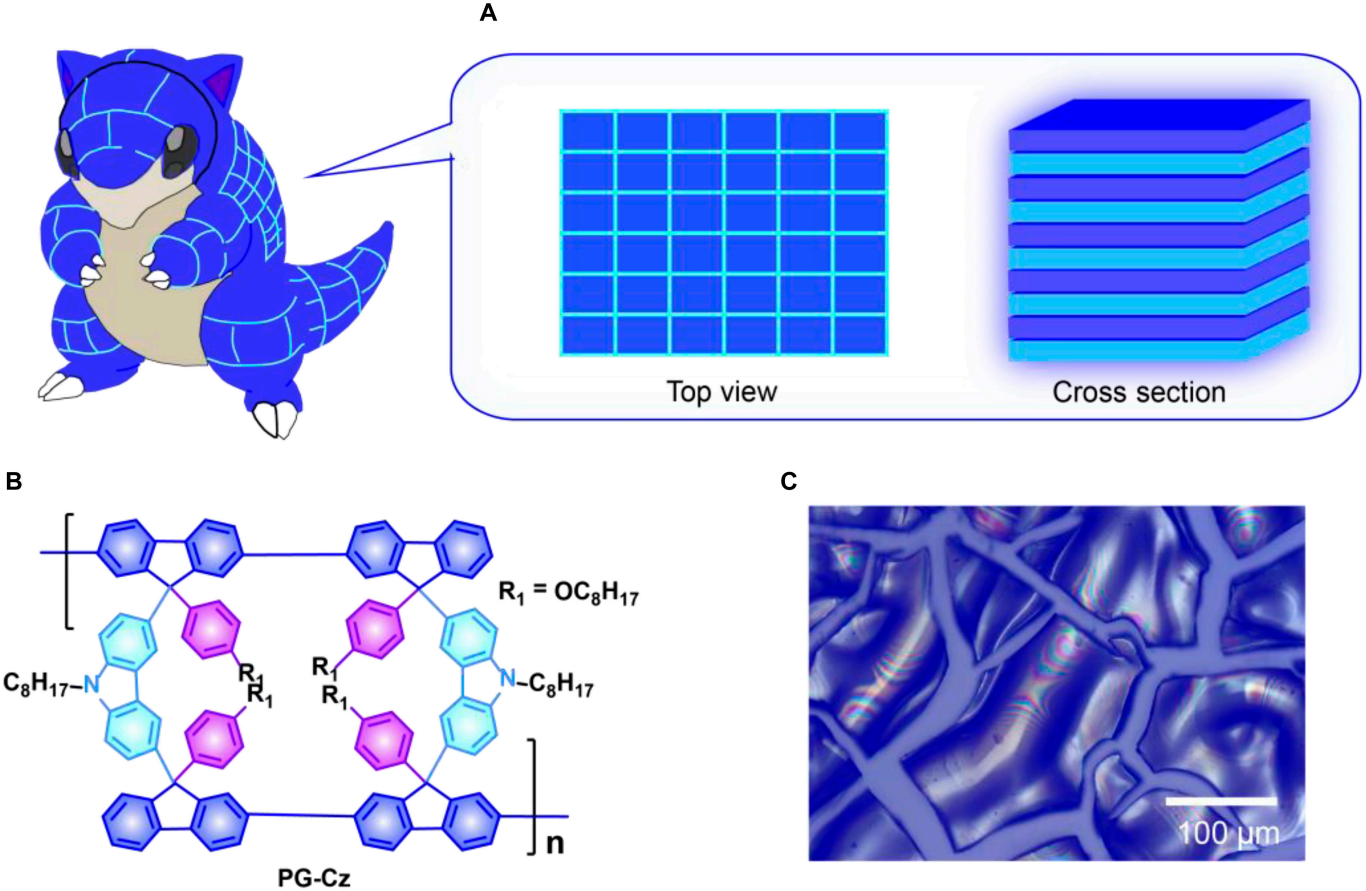
The molecular design of π-interrupted nanopolymers for multilayer crack films. (A) Inspired by the pangolins’ scaly skin. The sky-blue region is represented as the interface cracks. (B) Molecular structures of PG-Cz polygrid [38]. (C) The image of the interface cracks through the drop-casting process.

Gridarene-based π-nanopolymers (namely, polygrids) are the rising one-dimensional organic semiconductors with extensive tailorability in single-chain structure [34,35] and interchain association behaviors [9]. Polygrid chains with rigid rod-like conformation enable the achievement of high dielectric constants and ultralow energy disorder for excellent electrical performances [35]. Furthermore, PhC membranes with ultralow waveguide loss (2.6 cm^−1^) are afforded by the solution self-assembly of fully π-conjugated nanopolymers [9]. However, random lasing has not been observed, possibly due to insufficient optical feedback on the whole optical membrane. Herein, we innovatively induce the direct self-assembly of π-interrupted polygrids into complex morphologies integrating well-ordered multilayer structures with pangolin-skin-like interface cracks (Fig. 1A). This kind of polygrids consists of carbazole-bridged ladder-type gridarene units with covalent linkage in the diagonal direction (called PG-Cz, Fig. 1B). The π-interrupted backbones probably have a weaker interchain association and entanglement strength, which favorably generate cracks as the refractive-index inhomogeneity (Fig. 1C). By combining interface cracks with photonic-crystal-like multilayer structures, we establish a deep-blue random lasing with narrow linewidths and high-quality factors *Q* = 5,500 to 6,200. In addition, the quantum yields of deep-blue emission are relatively enhanced to *Φ* = 40% to 50% under micrometer-thick multilayer cracks. This work establishes a π-nanopolymer toolbox to tune complex morphologies and achieve organic high-*Q* random lasers, which has potential in the development of solution-processable optoelectronic circuits and electrically pumping lasers.

## Results

### Simulation of polygrids’ self-assembly

**Fig. 2.**
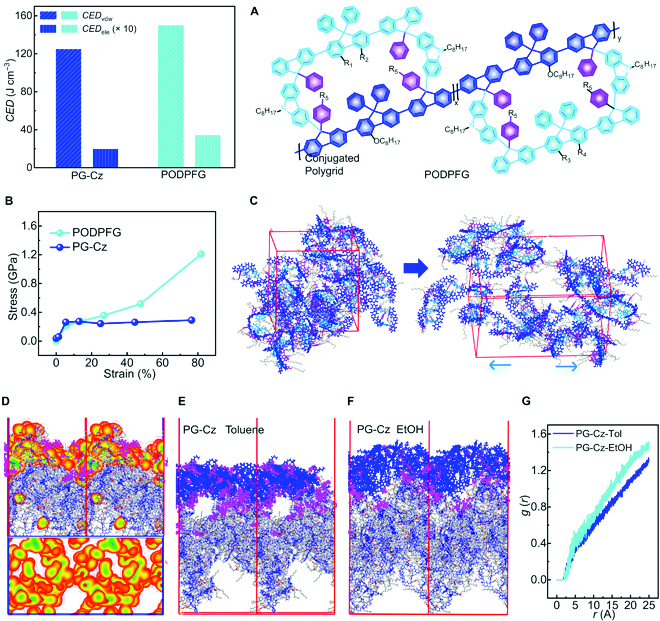
The molecular dynamics simulation of self-assembled microstructures. (A) The calculation of cohesive energy density (*CED*_vdw_ for the van der Waals part and *CED*_ele_ for the electrostatic interaction part) of PG-Cz and conjugated polygrid PODPFG (shown in molecular structure, corresponding values in our previous work [9]) aggregates. (B) The simulated stress–strain curve of PG-Cz-based and PODPFG-based aggregates, where the conformational change of PG-Cz is shown in (C). (D) Molecular dynamics simulations of toluene solvents on the deposited PG-Cz aggregate surface (marked in gray regions). The orange mapping is the distribution of toluene (the top view in blue boxes). (E and F) Molecular dynamics simulations of PG-Cz-toluene solution and PG-Cz EtOH solution, respectively. PG-Cz in solution is marked in blue and the specific solvent molecules are marked in purple. The radical distribution function between the deposited surface and the PG-Cz chains in solution is shown in (G).

Before the experimental investigation, we performed molecular dynamics simulations of PG-Cz aggregates to predict the formation of multilayer cracks. The conjugated polygrid poly{[4-(octyloxy)-9,9-diphenylfluoren-2,7-diyl]grid}-co-{[5-(octyloxy)-9,9-diphenylfluoren-2,7-diyl]grid} (PODPFG) [9] (Fig. 2A), which rarely in situ generates cracks, is also established as the control nanopolymer. Both PG-Cz and PODPFG aggregates, with the same degree of polymerization (*DP* = 10) for each single chain, are constructed in a thermodynamic equilibrium state. Primarily, the direct formation of cracks arises from the stress-induced interchain disentanglement [36,37], which easily occurs in aggregate systems with the low interchain association and weak entanglement strength, converse to self-healing polymers. In Fig. 2A, the PG-Cz aggregate displays a lower cohesive energy density (van der Waals part *CED*_vdw_ = 125 J m^−3^ and electrostatic part *CED*_ele_ = 1.99 J m^−3^) than that of PODPFG (*CED*_vdw_ = 150 J m^−3^ and *CED*_ele_ = 3.50 J m^−3^), suggesting the weaker interchain aggregation from π-interrupted nanopolymers. Meanwhile, the smaller radical distribution function in the PG-Cz aggregate (Fig. S1) also confirms the larger interchain distance. These results verify the presence of a stronger super-steric hindrance effect on PG-Cz chains. Furthermore, we simulated the stress–strain curve (Fig. 2B) to investigate the interchain entanglement strength. As a control simulation, PODPGF aggregate tolerates the tensile strain from 10% to 80%, where the interchain entanglement behaviors can be sustained under enhanced stress from 0.3 to 1.2 GPa (without mechanical fracture, Fig. S2). In contrast, the PG-Cz aggregate causes mechanical rupture under an ultimate strain of ~8%, where the tensile stress is unchangeable at 0.3 to 0.35 GPa. Figure 2C also reveals that the PG-Cz aggregate is readily disentangled into single chains under 0.3 to 0.35 GPa, revealing the much weaker strength of interchain entanglement. On this basis, we also calculated microscopic stresses of polymer–solvent coexisting systems (Fig. S4), which are likely derived from intermolecular interactions and molecular motions. In the coexisting systems of PG-Cz and toluene, microscopic stresses of 0.15 to 0.30 GPa (accounting for 27%) approximate the stress threshold of its disentanglement behavior (~0.30 GPa, as the start of mechanical rupture), while stronger stresses of 0.30 to 0.50 GPa (accounting for 9%) reach the disentanglement threshold. Thus, partly interchain disentanglement of the PG-Cz aggregate can occur for the generation of crack regions, converse to the PODPFG aggregate with lower microscopic stresses (0 to 0.5 GPa) than its disentanglement threshold (≥1.2 GPa).

We further simulated the self-assembly behaviors of PG-Cz to predict the feasibility of forming multilayer structures, based on the established aggregate structure as the deposited substrate. Such deposited aggregate displays a relatively rough surface with vacancy sizes of 1 to 3 nm, due to the presence of bulky repeat units and torsional main-chain conformation (confirmed by Raman spectra, Fig. S5). When adding a toluene drop onto the deposited surface, toluene molecules are diffused swiftly to reduce the free volume of vacancy (Fig. 2D and Fig. S6), owing to the strong solvation effect on PG-Cz chains (Fig. S9). In this case, other PG-Cz chains (diffused in solution) cannot occupy these vacancies even after a long simulation time of 2,400 ps (Fig. 2E and Fig. S10). After the evaporation of the toluene solvent, these vacancies can be residual as the air layer to facilitate the formation of multilayer structures. To investigate the solvent role in self-assembled microstructures, we replaced toluene with ethanol-type poor solvent (EtOH) to impair the solvation effect (Figs. S14 and S15) and found that PG-Cz chains can fill these vacancies (near the simulation time of 2,160 ps, Fig. 2F and Fig. S17). The larger radial distribution function at the aggregate interface in the EtOH solution (Fig. 2G) also confirms the closer interchain aggregation without vacancies. Therefore, the toluene-triggered solvation effect is fundamental to the in situ generation of multilayer architectures from PG-Cz aggregates.

### Demonstrations on self-assembled microstructures

**Fig. 3.**
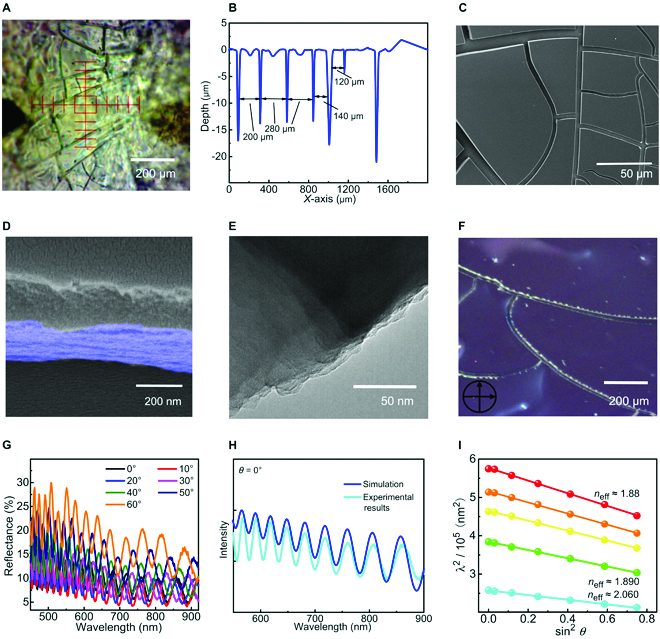
Structural demonstration of multilayer cracks. (A) The profile image of the PG-Cz-based solvent-casting film surface, along with the height profile shown in (B). (C) Scanning electron microscopy image of the PG-Cz-based solvent-casting film surface. Its cross-sectional image is shown in (D), along with images of high-resolution transmission electron microscopy in (E). (F) Polarized optical microscopy image of the PG-Cz-based solvent-casting film under the polarized light of 90°. (G) The angle-dependent reflectance spectra of the PG-Cz-based solvent-annealing film. The simulation of optical interference fringe at the incident angle *θ* = 0° is shown in (H). The demonstration of Bragg–Snell diffraction fitting is shown in (I), along with the effective refractive index (*n*_eff_) within the individual wavelength (*λ*) region.

To validate the above prediction from the molecular dynamics simulation, we investigated the self-assembly laws of PG-Cz through general solution-processing methods. The PG-Cz chains with a number-average *DP* of 13.3 were prepared in ~60% yields via the Yamamoto polymerization of ladder-type gridarene monomers (Fig. S20), according to our previous work [38]. Toluene solvent was selected during drop-casting, solvent-casting, and solvent-annealing processes, consistent with simulation models. The self-assembled microstructures were characterized by the profile method, scanning electron microscopy (SEM), high-resolution transmission electron microscopy (HR-TEM), and polarized optical microscopy (POM). Through the solvent-casting method, we did observe massive cracks with a width of 5 to 15 μm on the film surface, which were generated during the evaporation of toluene solvent. These cracks can divide the whole film into discrete microstructures with squares ranging from 10^3^ μm^2^ to 10^5^ to 10^6^ μm^2^ (in Fig. 3A to C), compared to the suitable sizes of microcavities. Deeply, we magnified the image of interface cracks (SEM images in Fig. 3D and HR-TEM images in Fig. 3E) and observed well-ordered multilayer architectures with close interlayer stacking like DBR cavities. Moreover, POM images show an optical anisotropy on the edges of interface cracks (Fig. 3F). As the probability of crystalline state is ruled out by selective-area electron diffraction and x-ray diffraction characterizations (Figs. S21 and S22), such optical anisotropy indicates the well-ordered orientation in multilayer structures. Apart from the solvent-casting method, the ultrafast drop-casting process (in ~5 min) can also form PG-Cz-based multilayer morphology with crack morphologies (Figs. S23 to S25), after completely evaporating toluene solvents. These results firmly support our hypothesis from the molecular dynamics simulation. In addition, the compact multilayer morphology can also be achieved through the solvent-annealing method (Figs. S26 to S29) from PG-Cz-based microsphere films, although relatively fewer cracks were generated.

We further demonstrated the multilayer crack morphologies through angle-resolution reflectance spectra (within wavelength *λ* = 450 to 900 nm). In Fig. 3G, the PG-Cz-based solvent-annealing film (without obvious interface cracks) shows a series of well-ordered interference fringe patterns that are blue-shifted by increasing the incident angle *θ* from 0° to 60°. Via simulating the interference fringe (Fig. 3H and Fig. S30), the periodic optical path difference from each layer is roughly evaluated as 6 nm (total thickness of 3.5 to 3.6 μm, ~600 layers), which is approximately consistent with the structural model in the molecular dynamics simulation. Meanwhile, the count mode spectra also show angle-dependent fringe peaks (Fig. S31) with a linear relationship sin^2^ *θ* ~*λ*^2^ (Fig. 3I), perfectly obeying the Bragg–Snell diffraction law of PhCs [39]. These results, which are also observed from PG-Cz-based solvent-casting and drop-casing films (Figs. S32 and S33), firmly confirm the mesoscale ordering in multilayer microstructures. Nevertheless, cracks on drop-casting films enable to diminish well-ordered optical interference, probably because of the enhancement in photonic scattering (Fig. S34, confirmed by lower optical transparency). Via extracting from the Bragg–Snell diffraction equation, the PG-Cz-based drop-casting film (with more interface cracks) shows a drastically lower efficient refractive index *n*_eff_ = 1.17 (Fig. S33) than that of the solvent-annealing film (*n*_eff_ = 1.88 to 1.89 for *λ* = 760 to 550 nm and *n*_eff_ = 2.060 for *λ* = 508 to 461 nm). The above results suggest the presence of air layers (*n* ≈ 1) in the crack regions. Notably, these interface cracks can serve as a natural refractive-index inhomogeneity (the refraction index contrast ∆*n* ≥ 0.8) to facilitate photonic reflection and lasing action along the in-plane direction of the film surface.

### Deep-blue emission with the enhancement of quantum yields

**Fig. 4.**
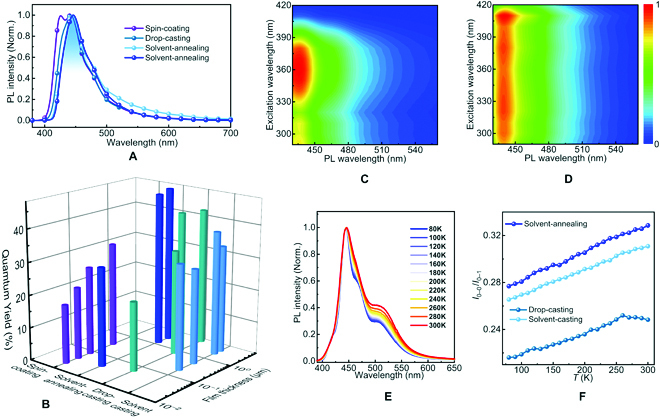
Luminescence features of PG-Cz-based multilayer crack films. (A) Photoluminescence spectra (PL) of PG-Cz-based spin-coating film, drop-casting film, solvent-casting film, and solvent-annealing film. (B) The quantum yield (*Φ*) of PG-Cz in the above various film states with various thicknesses. (C and D) The 2-dimensional PL spectra of the PG-Cz-based drop-casting film with small and large thicknesses, respectively. (E) Temperature-dependent PL spectra of the PG-Cz-based drop-casting film, under the temperature range of 80 to 300 K. The relative intensity of 0–0 emission to 0–1 emission (denoted as *I*_0–0_/*I*_0–1_) is extracted and provided in (F).

We characterized the photoluminescence features of PG-Cz-based multilayer cracks from drop-casting, solvent-casting, and solvent-annealing methods. In Fig. 4A, the multilayer crack films with 1 to 13 μm thicknesses exhibit the dominant emission at 445 to 450 nm (0–1 emission), while the 0–0 emission at 426 nm (observed in spin-coating thin films) is diminished. Moreover, multilayer crack films do not additionally exhibit green-band emission at 530 to 560 nm [40] even under micrometer-scale thickness, indicating the ultra-stable deep-blue emission with the sustained International Commission on illumination (CIE) coordination at (0.16, 0.10) (Fig. S35). The quantum yields of these multilayer crack films were calculated as *Φ* = 20% to 50%. Furthermore, these *Φ* values can be surprisingly elevated from 20% to 30% to 40% to 50% if the thickness of multilayer films is increased from 40 nm to at least 1.1 μm (Fig. 4B). For example, the multilayer microstructures from the solvent-annealing method show increased quantum yields from 30% to 47% to 48% when increasing the thicknesses from 40 nm to 1.3 μm and 2.5 μm, respectively. Similarly, the quantum yields of multilayer crack films via the drop-casting method are elevated from 21%, 32%, to 43% with a higher thickness (from 60 nm, 750 nm, to 1.1 μm, respectively), and can be maintained as *Φ* = 39% to 42% under thicknesses of 4.5 to 13 μm. In 2-dimensional PL spectra, the single excitation at 360 nm [38] (observed from thin films, Fig. 3C) can be assigned to the excitation on PG-Cz single chains, while the long-wavelength excitation peaks at 380 and 410 nm (observed from thick films with higher *Φ* values, Fig. 3D) can be assigned to the excitation on interchain aggregates. These results suggest that the enhancement of quantum yields is likely related to the interchain aggregation behaviors, contradictory to the feature in aggregation-caused quenching.

In addition, as the mode volume is too large (hundreds of micrometric scale) to induce a strong Purcell effect, the factor of microcavity effect [41] from multilayer structures is ruled out for the enhancement of quantum yields. Meanwhile, the microsphere architectures also increase the quantum yields (Fig. S44), further confirming the negligible influence of multilayer microstructures on emission enhancement. Moreover, the conformation of PG-Cz single chains is maintained in a nonplanar and torsional state (Fig. S5) rather than a planarized conformation state (as a fundamental role in increasing the quantum yields [18,42]), ruling out the possibilities from single-chain conformations. Thus, the above results also confirm that such emission enhancement is more probably linked with the interchain aggregation of PG-Cz.

Furthermore, temperature-dependent photoluminescence spectra (Tem-PL) are used to investigate the aggregate emission feature of PG-Cz in multilayer crack morphologies. In Fig. 4E, PG-Cz-based multilayer crack films via the drop-casting process show the dominant 0–1 emission at 443 nm and the rather weak 0–0 emission at 423 nm, both of which are not blue-shifted when elevating the temperature from 80 to 300 K. Moreover, the intensity ratio of 0–0 to 0–1 emission (*I*_0–0_/*I*_0–1_) can be relatively increased when elevating the temperature (Fig. 4F). The above findings, which are also observed on solvent-casting and solvent-annealing films (Fig. S45), verify the feature of H-aggregate emission [43]. Notably, such aggregate emission is drastically distinguished from the J-aggregate emission of PODPFG-based multilayer films, which exhibits lower quantum yields (*Φ* = 7% to 20%) on thick multilayer films (Fig. S47). Thus, the enhancement of quantum yields on multilayer crack films can be closely related to the H-aggregate emission of PG-Cz chains. In addition, the *I*_0–0_/*I*_0–1_ ratio of the drop-casting film (*I*_0–0_/*I*_0–1_ = 0.21 to 0.25) is slightly lower than that of solvent-casting (*I*_0–0_/*I*_0–1_ = 0.26 to 0.31) and solvent-annealing films (*I*_0–0_/*I*_0–1_ = 0.28 to 0.33) in the temperature range of 80 to 300 K, revealing the higher order of H-aggregate emission from the drop-casting method.

### Random lasing from multilayer cracks

**Fig. 5.**
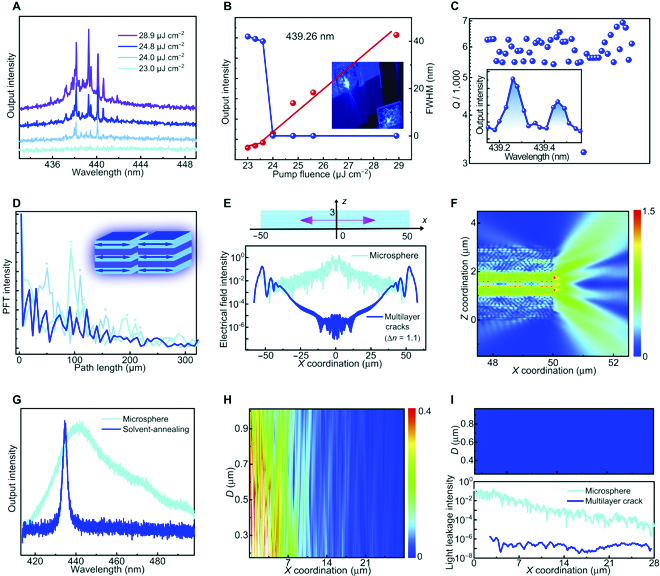
Investigation of the relationship between multilayer cracks and random lasing. (A) The output intensity of lasing on the PG-Cz-based drop-casting film. The calculation of lasing thresholds is shown in (B), along with the images of deep-blue lasing from the PG-Cz-based drop-casting film. (C) The calculation of *FWHM* and *Q*-factors (about 50 lasing peaks). (D) Power Fourier transform (PFT) spectra of lasing from drop-casting films with various cracked regions. (E) The simulated electric field intensity (100-μm sizes) in PG-Cz-based multilayer crack and microsphere films, through the finite-different time domain method. The distribution of the electric field (light source at *x* = 0) of the multilayer crack is shown in (F). (G) Luminescence spectra of the PG-Cz-based solvent-annealing film and microsphere film, under an energy density of 129.1 μJ cm^−2^. (H and I) The detected light leakage intensity mapping (the distance *D* from film regions is initially set as 0.2 μm) of microsphere film and multilayer crack, respectively. The light source is set at *x* = 1 to 2 μm.

We examined the application of multilayer crack morphologies in lasing actions, through 343-nm pulse pumping in the air. A pulse length of 1 ns, which is 3 to 4 times longer than the average fluorescence lifetimes (Fig. S40), was selected for a quasi-continuous-wave mode [44]. In Fig. 5A, the PG-Cz-based drop-casting film exhibits a series of narrow peaks at 438.15, 439.28, 440.13, and 440.61 nm, close to 0–1 emission in a 4-level system. The output intensity is nonlinearly increased along with increasing the energy density from 22.8 to 28.9μJ cm^−2^, and the threshold is achieved as 23.5 μJ cm^−2^ (Fig. 5B and S49). The full width at half maximum (FWHM) is measured as 0.07 to 0.08 nm (Fig. 5C), reflecting the existence of high temporal coherence. Meanwhile, we also observed the blue-emissive output beam with some degree of divergence (Fig. 5B). The above findings confirm the characteristics of random lasing actions [45] (with a gain coefficient of 37 cm^−1^, Fig. S50). The quality factor of random lasers can be up to *Q* = 5,500 to 6,200 through statistically evaluating about 50 emission peaks, according to the equation *Q* = *λ*/FWHM (Fig. 5C and Figs. S51 and S52). It is noted that even with the random nature of the crack formation, the ultranarrow linewidths (0.07 to 0.08 nm) and high-*Q* features (*Q* > 5,000) can also be maintained, which exceeds general random lasers (FWHM ≥ 0.5 nm, *Q* < 1,500) of conjugated polymers [18,19].

On this basis, we get insight into the likely mechanism between high-*Q* lasing actions and multilayer crack morphologies. Through power Fourier transform (PFT) of lasing spectra (Fig. 5D), the fundamental PFT peaks are distributed at the path length *d* = 18 to 100 μm, approximately corresponding to the cavity length (*L*_c_) of 50 to 300 μm according to the equation *L*_c_ = 2π*d*/*n*. These cavity sizes are roughly within or consistent with the length of discrete multilayer films between two crack interfaces, reflecting the in-plane direction of lasing oscillation (perpendicular to the multilayer cracks). Furthermore, we performed the finite-different time domain (FDTD) simulation to confirm the role of crack regions in optical behaviors. In Fig. 5E and F, the field intensity is sharply enhanced by at least 3 orders of magnitude near cracked interfaces, probably due to strong diffraction behaviors at the interface cracks with a large refractive index contrast (Figs. S53 to S55). As a control experiment, we also prepared the PG-Cz-based solvent-annealing film without obvious interface cracks, and we merely observed the amplified spontaneous emission (ASE) behavior with a linewidth of 3.4 to 3.8 nm, a threshold of ~16 μJ cm^−2^, and a gain coefficient of 25 cm^−1^ (Fig. 5G and Figs. S50, S56, and S57). These results confirm the key role of cracks in favoring optical gain behaviors and lasing actions.

Apart from interface cracks, we further investigated the effect of multilayer structures on such lasing action. The microsphere-deposited film (with nanoparticle sizes of 1 to 2 μm) was prepared as the controlled morphology, which also exhibits massive material/air interfaces like cracks but does not have mesoscale-ordered multilayer architectures (verified in Figs. S58 to S60). In Fig. 5G, the PG-Cz-based microsphere film does not perform lasing and ASE actions, even under the highest energy density of 129.1 μJ cm^−2^. The FDTD simulation reveals that the microsphere film displays an insufficient waveguide effect within the internal structure, and the luminescence enhancement at two sides of the edges is not observed (Fig. 5E and Fig. S61). In Fig. 5H, these features are attributed to the serious light leakage from strong light scattering on rough microsphere films, which cannot ensure a sufficient length of optical mean free path [13]. Conversely, the multilayer stacking film displays at least 3 orders of magnitude lower scattering and light leakage intensity under the same simulated distance from the film regions (as in microsphere cases, in Fig. 5I), supporting the low-loss optical waveguide (Fig. 5E) and feedback actions. Therefore, such random lasing originates from the ingenious interplay between crack-enhanced refractive index inhomogeneity and the multilayer-driven low-loss waveguide effect, which will establish creatively amorphous microlasers with low-cost and large-scale solution processing.

## Discussion

In conclusion, we have modulated a complex morphology of multilayer cracks from π-interrupted polygrid PG-Cz and achieved a deep-blue random laser simply through the drop-casting method. The molecular dynamics simulation reveals that PG-Cz exhibits weaker interchain aggregation and entanglement strength to promote the formation of interface cracks, and the toluene-induced solvation effect maintains nanoscale vacancies as the air layer of photonic-crystal-like multilayer microstructures. Furthermore, the modulation of H-aggregate emission allows PG-Cz to suppress concentration quenching for high-efficient deep-blue emission. Random lasing with high *Q* ≈ 5,500 to 6,200 is achieved by multilayer crack morphologies, with synergizing crack-induced refractive index inhomogeneity with the multilayer-triggered low-loss waveguide. These polygrids and high-*Q* random lasers will serve as the key organic semiconductor platform to realize the long-pursued target of organic electrically pumping lasers. The above findings will also provide an effective molecular toolbox for the simplification of solution-processing in developing organic on-chip photonics and lasing communications.

## Materials and Methods

### Preparation of polygrids and multilayer crack films

PG-Cz and PODPFG were prepared according to our previous work [9,38]. PG-Cz solution (with toluene solvent, using the high concentration of 5 to 20 mg ml^−1^) was prepared, and then the drop-casting (dropping solution onto the quartz substrate and then evaporating toluene solvent in 5 min), solvent-casting (transferring into plant vessel and slowing evaporating toluene solvent in 4 days), and solvent-annealing methods (depositing the PG-Cz-based microsphere film onto the quartz substrate under filling toluene atmosphere in 1 to 2 days) were used for the self-assembly into multilayer crack films, similar to our previous work [9].

### Molecular dynamics simulations

All molecular dynamics simulations are performed through the Forcite modules in the Material Studio 2016. For the simulation of aggregation models, 4 PG-Cz chains (with *DP* = 10 on each single-chain) are constructed in an amorphous cell to perform molecular dynamics simulation under a number–pressure–temperature constant (NPT) ensemble (fixed with 298 K temperature and 10^−4^ GPa pressure) and COMPASS force field (1.5 fs time step, 2,500 ps total time, and a cutoff distance of 15.5 Å for the summation of both van de Waals and electrostatic terms). Then, we perform the NPT dynamics simulation of PG-Cz aggregates under 298 K temperature, 1.5 fs time step, and 1,500 ps total time, in which each conformation information (such as the cohesive energy density and radial distribution function at 1,500 ps) is extracted at the interval of 7.5 ps. For the simulation of PG-Cz chains with toluene or ethanol solvent molecules, we establish corresponding amorphous cells with merely PG-Cz single chains (each with *DP* = 4, 2 chains), merely toluene or ethanol molecules (with 40 molecules), and the mixing systems with PG-Cz chains (each with DP = 4, 2 chains) and solvent molecules (toluene or ethanol, with 40 molecules), each of which was set at a density of 0.4 g cm^−3^. All of the above systems undergo geometric optimization under the same conditions as those of PG-Cz aggregates (SMART algorithm). Then, NVT dynamics simulation was performed under the COMPASS force field (298 K temperature, 1.5 fs time step, and 900 ps total time). For the molecular dynamics simulation of self-assembly at the interface (NVT ensemble). The time step is set as 1.5 fs, and the total time is set as 3 ns, where each conformational information is extracted at the interval of 12 ps. For the simulation of stretching polygrid entanglement aggregate (using Forcite models), the input stress is set as 0, 2, 4, 6, 8, 10, and 12 GPa, respectively, along with the NPT ensemble, Souza-Martins Barostat, and Nose-Hoover-Langevin thermostat. The calculation of microscopic stress on each plane direction (200 data of conformational states for each plane of *XY*, *YZ*, and *XZ*) is performed under the NVT ensemble.

### Structural demonstrations

The GPC spectral of PG-Cz oligomers and long single chains were obtained through an HP1100 HPLC system equipped with 7911GP-502 and GPC columns (polystyrene as the standard and tetrahydrofuran as the eluent). The detection of conformational states in self-assembled solid is based on the Raman spectra with a Horiba evolution spectrometer, which is coupled to an Olympus BH-2 confocal microscope (the excitation source is based on the He–Ne laser with the 633-nm line). The microspheres of PG-Cz-based microsphere films were observed by an inverted fluorescence microscope 278 (FV1000-IX71, Tokyo, Japan), as a demonstration of its blue-emitting feature. The large-scale morphologies are observed by profile (Bruker) and SEM (Hitachi, S-4800, under the condition of 5-kV accelerating voltage, deposited onto silicon wafer followed by the gold-spraying treatment). TEM (Hitachi, HT7700, under the conditions of ~10-kV accelerating voltage) and HRTEM (FEI, Talos F200X, with the 200-kV accelerating voltage) were also applied to further testify the self-assembled morphologies and selected-area electron diffraction. X-ray diffraction was performed on a Bruker D8 x-ray diffractometer with Cu KR radiation (*λ* = 1.54050 Å). The highly ordered multilayer microstructures of PG-Cz-based solvent-casting and solvent-annealing films were also examined by the POM characterizations with NIKON LV100ND systems.

### Optical characterizations and simulation

The light–matter interaction of these multilayer crack morphologies was characterized by the angle-resolved spectrum system with micro-region (ARMS-U2, ideaoptics, China). The visible light source (with emissive wavelength centered at 550 to 750 nm) is the halogen lamp. We used the constant angle of incidence and reflection mode as the test mode, during which the incidence angle (*θ* = 0° to 60°) is identical to the detected reflectance angle. The aluminum mirror serves as the standard. The elimination of the background scattering is based on the black sponge, which transforms the count mode spectra into the reflectance spectra with absolute reflectance values. In addition, the transmittance (*T*_o_) of various PG-Cz-based films is obtained through the transformation from the absolute absorbance (*A*_o_), according to the equation *A*_o_ = −lg *T*_o_. The absorbance can be obtained via the ultraviolet spectra from LAMBDA 35 systems. The simulation of optical interference fringe is performed by MATLAB software (note that the absolute intensity cannot be set the same as the experimental light source; thus, the intensity cannot be fitted well, but the interference fringe fitting wavelength can be realized among all 0° to 60° cases).

### Luminescence characterizations

The deep-blue emission and 2-dimensional PL spectra of various PG-Cz-based films were detected through RF-6000 Plus systems. The *Φ* values of deep-blue emission on various PG-Cz-based films were examined by an Edinburgh FLSP920 fluorescence spectrophotometer with a xenon arc lamp (Xe900). The integral sphere was used to collect the photons including the reflected types from the xenon arc lamp and the emissive types from PG-Cz chains. The *Φ* values can be evaluated by the ratio of emissive (*I*_e_, defined as the integration of intensity within the whole sample-contained emission spectra in the range of 380 to 680 nm) to absorbed (*I*_a_) numbers of photons from PG-Cz chains, where the *I*_a_ values can be obtained by the differences of reflected photons between the quartz background (*I*_b_, defined as the integration of intensity within scattering spectra of quartz background under the excitation wavelength of 350 to 360 nm) and the film samples (*I*_s_, defined as the integration of intensity within scattering spectra of samples under the excitation wavelength of 350 to 360 nm). Thus, the *Φ* values can be obtained via the equation *Φ* = *I*_e_/(*I*_b_ − *I*_s_). To improve the testing accuracy of collected photons, all processes of the photonic collection were performed by repeating 2 cycles. The fluorescence lifetime of various PG-Cz-based films was detected by the spectrometer system (Princeton Instrument Acton SP2500I) that is equipped with a streak camera (Optronis Optoscope sc-10), based on the excitation through a 325-nm femtosecond pulse laser. Tem-PL was employed by the 355-nm diode laser (from Changchun New Industries Optoelectronics Tech. Co., Ltd. MDL-III-447L, along with the detecting systems through Idea Optics PG-4000 spectrometer) to afford the photoluminescence spectra under the environment temperature varying from 80 to 300 K, where the liquid nitrogen cryostat was used to maintain the environment at ultralow temperature (80 K).

### Random lasing characterizations

The pump source (Coherent Inc., Santa Clara, CA, USA) with an excitation wavelength of 343 nm, a pulse width of 1 ns, and a repetition rate of 100 Hz were selected for detecting random lasing. The spectra of ASE and lasing are detected by an optical spectrometer (HR 4000, Ocean Optics) that exhibits a resolution of ~0.03 nm. The gain coefficient of ASE and lasing is based on the variable stripe length measurement.

### Simulation of electromagnetic field distribution and light leakage intensity

The FDTD simulation is performed, where the simulation is based on the *X*–*Z* coordination. The multilayer crack film is set as the model with a length of 100 μm (covering the *X* coordination of −50 to 50 μm) and a total height of 3 μm (with 500 layers, each layer has a 5-nm film and a 1-nm air layer). The refractive index of the film region is set as *n* = 2.1 according to the angle-resolution reflectance spectra. On 2 sides of interface cracks, the virtual region (with a length of 10 μm and a height of 3 μm, covering the *X* coordination of 50 to 60 μm and −60 to −50 μm, respectively) is added with a tunable refractive index (*n* = 1.0, 1.6 and 1.9) to investigate the influence of refractive index contrast of interface cracks on-field intensity at the ends. The model of microsphere film is established by the same total sizes with 300 microspheres (on the *Z*-axis, 3 layers of microspheres are established and each layer has 100 microspheres, based on the diameter of 1 μm for each microsphere with setting the same refractive index of 2.1). The light source is selected with the wavelength centered at 439.3 nm (consisting of the dominant lasing or ASE or luminescence wavelength). The detection of light leakage intensity is based on the 2-dimensional monitor established above the film surface initially at 0.2 μm.

## Data Availability

All of the relevant data in this work are available upon request from the corresponding author under reasonable request.

## Supplementary Materials

Supplementary Materials Table S1. The cohesive energy density (*CED*) of PG-Cz aggregates. Table S2. The exact molecular weight (*M*) of PG-Cz oligomers as a variation of elution time (*t*). Table S3. The elution times of PG-Cz chains with specific *DP*. Fig. S1. The radical distributed function (*g* (*r*)) of PG-Cz and PODPFG. Fig. S2. Molecular dynamics simulation of stretching PODPFG-based entanglement aggregate. Fig. S3. Molecular dynamics simulation of stretching PG-Cz-based entanglement aggregate. Fig. S4. Microscopic stress of nanopolymer-based aggregate systems (in the form of pressure). Fig. S5. The Raman spectra of various PG-Cz-based films (including the spin-coating, drop-casting, solvent-casting, and solvent-annealing films). Fig. S6. Molecular dynamics simulation of toluene solvent dropped onto the deposited PG-Cz aggregate surface. Fig. S7. The molecular dynamics simulation of PG-Cz chains with toluene molecules. Fig. S8. The intermolecular interaction energy (*E*_inter_) between PG-Cz chains and toluene solvent molecules. Fig. S9. The *E*_inter_ values between PG-Cz chains and toluene. Fig. S10. Molecular dynamics simulation of PG-Cz solution dropped onto the deposited PG-Cz aggregate surface. Fig. S11. The single-chain end-to-end distance (*L*_e_) of PG-Cz chains as a function of time, with toluene or EtOH environment. Fig. S12. The interchain distance of PG-Cz chains in a toluene or EtOH environment. Fig. S13. The detection of intermolecular interactions between PG-Cz and EtOH solvent. Fig. S14. The *E*_inter_ values between PG-Cz chains and EtOH molecules. Fig. S15. The *E*_inter_ values between PG-Cz chains and EtOH. Fig. S16. Molecular dynamics simulation of EtOH solvent dropped onto the deposited PG-Cz aggregate surface. Fig. S17. Molecular dynamics simulation of PG-Cz solution dropped onto the deposited PG-Cz aggregate surface. Fig. S18. MALDI-TOF-MS of PG-Cz oligomers (in linear mode). Fig. S19. The GPC spectra of PG-Cz oligomers. Fig. S20. The GPC spectra of PG-Cz. Fig. S21. Selected-area electron diffraction pattern of PG-Cz-based multilayer crack from the solvent-casting method. Fig. S22. X-ray diffraction of PG-Cz-based multilayer crack from the solvent-casting method. Fig. S23. Surface morphologies of PG-Cz-based interface cracks from the drop-casting method. Fig. S24. The SEM images of the multilayer structure from the drop-casting method. Fig. S25. The TEM and POM images of multilayer cracks from the drop-casting method. Fig. S26. Morphological transformation of PG-Cz from microsphere films to multilayer crack morphologies through the solvent-annealing method. Fig. S27. Deep observation of morphological transformation of PG-Cz chains at 30 min. Fig. S28. Surface morphologies from solvent-annealing. Fig. S29. HR-TEM images of the PG-Cz-based solvent-annealing film. Fig. S30. The simulation of reflected interference fringe in the incident angle range of 10° to 60°. Fig. S31. The count mode spectra of the PG-Cz-based solvent-annealing film. Fig. S32. The angle-dependent reflectance spectra of the PG-Cz-based solvent-casting film. Fig. S33. The angle-dependent reflectance spectra of the PG-Cz-based drop-casting film. Fig. S34. The transmittance of various PG-Cz-based self-assembled films. Fig. S35. CIE coordination of various PG-Cz-based films with multilayer crack morphologies (1 to 10 μm thickness). Fig. S36. The measurements for the calculation of absolute fluorescence quantum yields on PG-Cz-based spin-coating films with varying thicknesses (20, 40, 80, and 320 nm). Fig. S37. The measurements for the calculation of absolute fluorescence quantum yields on PG-Cz-based solvent-annealing films with varying thicknesses (40 nm, 1.3 μm, and 2.5 μm). Fig. S38. The measurements for the calculation of absolute fluorescence quantum yields on PG-Cz-based drop-casting films with varying thicknesses (60 nm, 750 nm, 1.1 μm, 4.5 μm, and 13 μm). Fig. S39. The measurements for the calculation of absolute fluorescence quantum yields on PG-Cz-based solvent-casting films with varying thicknesses (200 nm, 500 nm, 2 μm, and 3 μm). Fig. S40. The fluorescence lifetime of various films. Fig. S41. The excitation spectra of PG-Cz-based solvent-annealing films with various thicknesses. Fig. S42. The excitation spectra of PG-Cz-based drop-casting films with various thicknesses. Fig. S43. The excitation spectra of PG-Cz-based solvent-casting films with various thicknesses. Fig. S44. The measurements for the calculation of absolute fluorescence quantum yields on PG-Cz-based microsphere films with varying thicknesses. Fig. S45. The temperature-dependent PL spectra of the PG-Cz-based solvent-casting film and solvent-annealing film, under the temperature range of 80 to 300 K. Fig. S46. The temperature-dependent PL spectra of microsphere films. Fig. S47. The measurements for the calculation of absolute fluorescence quantum yields on various PODPFG-based films. Fig. S48. The changes of random lasing intensity as the variation of energy density (22.7 to 28.9 μJ cm^−2^) on the PG-Cz-based drop-casting film with massive material/air interfaces. Fig. S49. The threshold calculation on other lasing emission peaks at 438.15, 440.15, and 440.61 nm. Fig. S50. The gain coefficient of ASE (from the solvent-annealing film) and lasing (from the drop-casting film). Fig. S51. Statistic calculation of quality factors among various lasing peaks. Fig. S52. Magnifying the lasing spectra in a range of 1-nm width. Fig. S53. The FDTD simulation on multilayer stacking films with ∆*n* = 1.1. Fig. S54. The FDTD simulation on multilayer stacking films with ∆*n* = 0.2. Fig. S55. The FDTD simulation on multilayer stacking films with ∆*n* = 0.5. Fig. S56. The changes of ASE intensity as the variation of energy density (18 to 50 μJ cm^−2^). Fig. S57. The threshold calculation of ASE action. Fig. S58. The SEM image of the PG-Cz-based microsphere film. Fig. S59. The fluorescence microscopy image of the PG-Cz-based microsphere film. Fig. S60. The angle-dependent reflectance spectra of the PG-Cz-based microsphere film. Fig. S61. The FDTD simulation on microsphere films.
