# Supplementary material for: In Situ Super-Hindrance-Triggered Multilayer Cracks for Random Lasing in π-Functional Nanopolymer Films
Source: Research (Wash D C). 2023 Jan 16;6:0027. doi: 10.34133/research.0027 (PMC10076025; doi:10.34133/research.0027)
Supplement: Supplementary Materials — Table S1. The cohesive energy density (CED) of PG-Cz aggregates. Table S2. The exact molecular weight (M) of PG-Cz oligomers as a variation of elution time (t). Table S3. The elution times of PG-Cz chains with specific DP. Fig. S1. The radical distributed function (g (r)) of PG-Cz and PODPFG. Fig. S2. Molecular dynamics simulation of stretching PODPFG-based entanglement aggregate. Fig. S3. Molecular dynamics simulation of stretching PG-Cz-based entanglement aggregate. Fig. S4. Microscopic stress of nanopolymer-based aggregate systems (in the form of pressure). Fig. S5. The Raman spectra of various PG-Cz-based films (including the spin-coating, drop-casting, solvent-casting, and solvent-annealing films). Fig. S6. Molecular dynamics simulation of toluene solvent dropped onto the deposited PG-Cz aggregate surface. Fig. S7. The molecular dynamics simulation of PG-Cz chains with toluene molecules. Fig. S8. The intermolecular interaction energy (Einter) between PG-Cz chains and toluene solvent molecules. Fig. S9. The Einter values between PG-Cz chains and toluene. Fig. S10. Molecular dynamics simulation of PG-Cz solution dropped onto the deposited PG-Cz aggregate surface. Fig. S11. The single-chain end-to-end distance (Le) of PG-Cz chains as a function of time, with toluene or EtOH environment. Fig. S12. The interchain distance of PG-Cz chains in a toluene or EtOH environment. Fig. S13. The detection of intermolecular interactions between PG-Cz and EtOH solvent. Fig. S14. The Einter values between PG-Cz chains and EtOH molecules. Fig. S15. The Einter values between PG-Cz chains and EtOH. Fig. S16. Molecular dynamics simulation of EtOH solvent dropped onto the deposited PG-Cz aggregate surface. Fig. S17. Molecular dynamics simulation of PG-Cz solution dropped onto the deposited PG-Cz aggregate surface. Fig. S18. MALDI-TOF-MS of PG-Cz oligomers (in linear mode). Fig. S19. The GPC spectra of PG-Cz oligomers. Fig. S20. The GPC spectra of PG-Cz. Fig. S21. Selected-area electro [file research.0027.f1.docx]

Supplementary Materials

In-Situ Super-Hindrance-Triggered Multilayer Cracks for Random Lasing in π-Functional Nanopolymer Films

Dongqing Lin^1^, Yang Li^1^, He Zhang^1^, Shuai Zhang^2^, Yuezheng Gao^1^, Tianrui Zhai^2*^, Shu Hu^3^, Chuanxiang Sheng^3^, Heng Guo^4^, Chunxiang Xu^4^, Ying Wei^1*^, Shifeng Li^5^, Yelong Han^1^, Quanyou Feng^1^, Shasha Wang^1^, Linghai Xie^1*^, Wei Huang^1,6*^

The super-hindrance effect of PG-Cz can be evaluated through the cohesive energy density (*CED*), which shows the attractive energy in each amorphous cell unit. The cohesive energy (*E*_c_) for the interchain aggregation part was calculated as the equation **ES1**:

*E*_c_ = - <*E*_inter_> = - (<*E*_total_> - <*E*_intra_>) **ES1**

Where <*E*_total_>, <*E*_intra_>, and <*E*_inter_> are defined as the total energy of the system, intramolecular and intermolecular energy, respectively. Using the absolute value of *E*_c_ (always with *E*_c_ < 0 that is defined as the energy of attractive noncovalent interactions). The calculation of *CED* values is referred to in the equation **ES2**:

*CED* = *E*_c_ / V  **ES2**

Where the V is denoted as the amorphous cell volume of PG-Cz aggregate. Notably, the higher *CED* value reflects the stronger intermolecular attractive interactions with more powerful attractive potential, which requires more energy to separate intermolecular aggregates into single chains. Thus, the higher CED value corresponds to the weaker steric hindrance effect with repulsive interactions. For the molecular dynamic simulation using COMPASS forcefield, the noncovalent interactions can be divided into the van der Waals force part and the electrostatic force part, where the strong dipole hydrogen bonds are also included. As a result, the *CED* value consists of two corresponding part: *CED*_vdW_ (for van der Waals interactions) and *CED*_ele_ (for electrostatic interactions).

**Table S1: The cohesive energy density (CED) of PG-Cz aggregates.** The volume of amorphous cells is calculated as 145 nm^3^ based on dynamic equilibrium under NPT and NVT ensembles.

| Frame | *CED*_vdW_  (J cm^-3^) | *CED*_ele_  (J cm^-3^) | Intermolecular van der Waals energy  (kcal mol^-1^) | Intermolecular electrostatic energy (kcal mol^-1^) |
| --- | --- | --- | --- | --- |
| 1 | 124 | 2.22 | -2587 | -46.42 |
| 2 | 125 | 2.07 | -2606 | -43.29 |
| 3 | 125 | 2.19 | -2624 | -45.82 |
| 4 | 126 | 2.15 | -2645 | -45.00 |
| 5 | 126 | 2.24 | -2631 | -46.89 |
| 6 | 125 | 2.27 | -2612 | -47.38 |
| 7 | 127 | 2.28 | -2647 | -47.62 |
| 8 | 124 | 2.29 | -2603 | -47.89 |
| 9 | 125 | 2.05 | -2607 | -42.96 |
| 10 | 125 | 1.83 | -2620 | -38.32 |
| 11 | 125 | 2.02 | -2621 | -42.24 |
| 12 | 125 | 2.00 | -2605 | -41.74 |
| 13 | 124 | 2.20 | -2601 | -45.99 |
| 14 | 126 | 2.17 | -2643 | -45.35 |
| 15 | 125 | 2.20 | -2623 | -45.94 |
| 16 | 126 | 2.23 | -2633 | -46.57 |
| 17 | 126 | 2.17 | -2633 | -45.34 |
| 18 | 125 | 2.25 | -2615 | -47.01 |
| 19 | 125 | 2.25 | -2620 | -47.03 |
| 20 | 124 | 1.73 | -2602 | -36.14 |
| 21 | 126 | 2.11 | -2636 | -44.06 |
| 22 | 126 | 2.15 | -2636 | -44.89 |
| 23 | 125 | 1.97 | -2621 | -41.29 |
| 24 | 124 | 2.15 | -2592 | -45.00 |
| 25 | 125 | 2.07 | -2616 | -43.34 |
| 26 | 126 | 2.25 | -2633 | -47.15 |
| 27 | 127 | 2.10 | -2646 | -43.86 |
| 28 | 125 | 2.14 | -2604 | -44.83 |
| 29 | 126 | 2.09 | -2642 | -43.66 |
| 30 | 125 | 2.19 | -2622 | -45.90 |
| 31 | 126 | 2.31 | -2635 | -48.21 |
| 32 | 124 | 1.99 | -2596 | -41.55 |
| 33 | 126 | 2.27 | -2627 | -47.53 |
| 34 | 126 | 2.69 | -2634 | -56.34 |
| 35 | 125 | 2.38 | -2610 | -49.80 |
| 36 | 125 | 2.13 | -2616 | -44.74 |
| 37 | 125 | 2.02 | -2621 | -42.20 |
| 38 | 125 | 2.02 | -2617 | -42.26 |
| 39 | 125 | 2.03 | -2615 | -42.56 |
| 40 | 126 | 2.15 | -2626 | -44.91 |
| 41 | 125 | 2.16 | -2609 | -45.14 |
| 42 | 125 | 2.06 | -2611 | -43.06 |
| 43 | 124 | 1.97 | -2596 | -41.26 |
| 44 | 126 | 1.96 | -2643 | -41.02 |
| 45 | 125 | 2.16 | -2609 | -45.08 |
| 46 | 124 | 2.37 | -2600 | -49.48 |
| 47 | 126 | 2.14 | -2625 | -44.73 |
| 48 | 124 | 1.96 | -2603 | -40.91 |
| 49 | 123 | 2.08 | -2567 | -43.43 |
| 50 | 125 | 1.77 | -2611 | -37.03 |
| 51 | 125 | 1.99 | -2608 | -41.72 |
| 52 | 125 | 2.14 | -2619 | -44.82 |
| 53 | 125 | 1.99 | -2610 | -41.52 |
| 54 | 127 | 1.95 | -2647 | -40.81 |
| 55 | 125 | 1.92 | -2614 | -40.24 |
| 56 | 125 | 2.32 | -2625 | -48.53 |
| 57 | 125 | 2.14 | -2604 | -44.69 |
| 58 | 124 | 2.00 | -2589 | -41.79 |
| 59 | 126 | 2.29 | -2627 | -47.80 |
| 60 | 124 | 2.12 | -2603 | -44.27 |
| 61 | 124 | 2.20 | -2588 | -46.06 |
| 62 | 125 | 1.96 | -2607 | -41.06 |
| 63 | 123 | 1.84 | -2575 | -38.58 |
| 64 | 124 | 2.15 | -2586 | -44.90 |
| 65 | 124 | 2.29 | -2593 | -47.80 |
| 66 | 123 | 2.08 | -2577 | -43.50 |
| 67 | 123 | 2.14 | -2573 | -44.83 |
| 68 | 124 | 2.13 | -2595 | -44.55 |
| 69 | 123 | 1.98 | -2577 | -41.38 |
| 70 | 125 | 2.03 | -2623 | -42.42 |
| 71 | 124 | 2.04 | -2595 | -42.77 |
| 72 | 125 | 2.21 | -2612 | -46.24 |
| 73 | 124 | 1.79 | -2603 | -37.43 |
| 74 | 124 | 2.00 | -2599 | -41.86 |
| 75 | 125 | 1.99 | -2605 | -41.52 |
| 76 | 124 | 2.26 | -2603 | -47.18 |
| 77 | 123 | 2.27 | -2581 | -47.56 |
| 78 | 125 | 2.22 | -2623 | -46.40 |
| 79 | 125 | 2.06 | -2606 | -43.17 |
| 80 | 124 | 1.61 | -2591 | -33.64 |
| 81 | 124 | 1.94 | -2593 | -40.55 |
| 82 | 125 | 2.25 | -2617 | -47.09 |
| 83 | 125 | 2.21 | -2609 | -46.13 |
| 84 | 124 | 2.14 | -2603 | -44.76 |
| 85 | 123 | 2.17 | -2583 | -45.37 |
| 86 | 125 | 2.26 | -2621 | -47.24 |
| 87 | 124 | 2.13 | -2588 | -44.59 |
| 88 | 123 | 1.81 | -2578 | -37.81 |
| 89 | 124 | 1.83 | -2595 | -38.36 |
| 90 | 125 | 2.00 | -2614 | -41.86 |
| 91 | 125 | 2.21 | -2596 | -46.16 |
| 92 | 126 | 1.77 | -2629 | -36.96 |
| 93 | 126 | 2.17 | -2615 | -45.49 |
| 94 | 125 | 2.19 | -2623 | -45.82 |
| 95 | 125 | 2.22 | -2613 | -46.44 |
| 96 | 124 | 2.18 | -2595 | -45.63 |
| 97 | 124 | 2.37 | -2588 | -49.57 |
| 98 | 125 | 2.07 | -2619 | -43.33 |
| 99 | 124 | 1.88 | -2599 | -39.33 |
| 100 | 124 | 2.38 | -2597 | -49.87 |
| 101 | 124 | 2.11 | -2602 | -44.18 |
| 102 | 123 | 2.26 | -2583 | -47.24 |
| 103 | 125 | 2.10 | -2618 | -43.96 |
| 104 | 125 | 2.15 | -2614 | -45.04 |
| 105 | 124 | 2.27 | -2601 | -47.38 |
| 106 | 123 | 2.30 | -2581 | -48.01 |
| 107 | 124 | 2.10 | -2589 | -43.85 |
| 108 | 124 | 2.12 | -2600 | -44.27 |
| 109 | 124 | 2.25 | -2601 | -46.97 |
| 110 | 124 | 1.97 | -2583 | -41.29 |
| 111 | 124 | 2.08 | -2560 | -43.55 |
| 112 | 125 | 2.20 | -2624 | -46.02 |
| 113 | 125 | 2.20 | -2609 | -46.02 |
| 114 | 125 | 1.63 | -2620 | -34.18 |
| 115 | 124 | 2.01 | -2592 | -42.12 |
| 116 | 125 | 2.15 | -2616 | -44.99 |
| 117 | 128 | 1.83 | -2671 | -38.33 |
| 118 | 126 | 1.83 | -2645 | -38.26 |
| 119 | 126 | 2.10 | -2627 | -43.88 |
| 120 | 124 | 1.95 | -2593 | -40.82 |
| 121 | 124 | 2.10 | -2601 | -44.02 |
| 122 | 124 | 1.70 | -2602 | -35.46 |
| 123 | 126 | 1.72 | -2635 | -35.96 |
| 124 | 126 | 1.65 | -2635 | -34.58 |
| 125 | 124 | 1.69 | -2587 | -35.30 |
| 126 | 125 | 1.44 | -2609 | -30.15 |
| 127 | 126 | 1.89 | -2639 | -39.47 |
| 128 | 123 | 1.98 | -2582 | -41.46 |
| 129 | 125 | 1.97 | -2616 | -41.29 |
| 130 | 124 | 1.81 | -2590 | -37.81 |
| 131 | 123 | 1.78 | -2576 | -37.21 |
| 132 | 124 | 1.41 | -2595 | -29.41 |
| 133 | 125 | 1.88 | -2605 | -39.35 |
| 134 | 125 | 2.09 | -2623 | -43.70 |
| 135 | 123 | 2.10 | -2574 | -43.97 |
| 136 | 124 | 1.94 | -2593 | -40.64 |
| 137 | 123 | 1.86 | -2582 | -38.81 |
| 138 | 124 | 1.71 | -2599 | -35.70 |
| 139 | 123 | 2.18 | -2565 | -45.54 |
| 140 | 126 | 1.95 | -2640 | -40.81 |
| 141 | 125 | 2.12 | -2612 | -44.27 |
| 142 | 127 | 1.75 | -2661 | -36.67 |
| 143 | 125 | 1.84 | -2614 | -38.52 |
| 144 | 125 | 1.92 | -2606 | -40.15 |
| 145 | 1.25 | 1.85 | -2606 | -38.75 |
| 146 | 124 | 1.99 | -2589 | -41.65 |
| 147 | 125 | 2.15 | -2611 | -45.03 |
| 148 | 123 | 1.73 | -2567 | -36.25 |
| 149 | 125 | 1.97 | -2610 | -41.26 |
| 150 | 122 | 1.83 | -2552 | -38.18 |
| 151 | 123 | 1.82 | -2580 | -38.05 |
| 152 | 124 | 1.44 | -2595 | -30.02 |
| 153 | 124 | 1.74 | -2604 | -36.35 |
| 154 | 125 | 1.74 | -2619 | -36.36 |
| 155 | 123 | 1.59 | -2577 | -33.20 |
| 156 | 125 | 1.84 | -2612 | -38.59 |
| 157 | 124 | 2.32 | -2591 | -48.47 |
| 158 | 126 | 1.76 | -2630 | -36.80 |
| 159 | 125 | 1.88 | -2604 | -39.26 |
| 160 | 125 | 2.06 | -2605 | -43.17 |
| 161 | 125 | 1.79 | -2609 | -37.51 |
| 162 | 124 | 1.66 | -2590 | -34.65 |
| 163 | 125 | 1.83 | -2615 | -38.30 |
| 164 | 123 | 1.72 | -2583 | -36.03 |
| 165 | 123 | 1.95 | -2582 | -40.78 |
| 166 | 123 | 2.19 | -2572 | -45.73 |
| 167 | 123 | 1.82 | -2581 | -37.99 |
| 168 | 123 | 1.91 | -2574 | -39.89 |
| 169 | 125 | 1.51 | -2610 | -31.49 |
| 170 | 124 | 1.90 | -2591 | -39.83 |
| 171 | 125 | 1.65 | -2620 | -34.49 |
| 172 | 124 | 1.73 | -2586 | -36.22 |
| 173 | 126 | 1.88 | -2640 | -39.23 |
| 174 | 126 | 1.55 | -2641 | -32.38 |
| 175 | 123 | 1.82 | -2580 | -38.05 |
| 176 | 125 | 1.65 | -2622 | -34.44 |
| 177 | 124 | 1.73 | -2602 | -36.25 |
| 178 | 125 | 1.74 | -2611 | -36.36 |
| 179 | 125 | 1.78 | -2611 | -37.25 |
| 180 | 126 | 1.89 | -2631 | -39.63 |
| 181 | 125 | 1.52 | -2606 | -31.73 |
| 182 | 124 | 1.55 | -2595 | -32.34 |
| 183 | 1.23 | 1.54 | -2567 | -32.11 |
| 184 | 123 | 1.58 | -2571 | -33.04 |
| 185 | 126 | 1.99 | -2626 | -41.53 |
| 186 | 125 | 1.69 | -2619 | -35.29 |
| 187 | 125 | 1.95 | -2624 | -40.75 |
| 188 | 126 | 1.64 | -2630 | -34.30 |
| 189 | 124 | 2.18 | -2592 | -45.63 |
| 190 | 124 | 1.74 | -2585 | -36.48 |
| 191 | 125 | 1.97 | -2616 | -41.14 |
| 192 | 125 | 1.67 | -2613 | -34.86 |
| 193 | 124 | 1.78 | -2596 | -37.19 |
| 194 | 126 | 1.66 | -2627 | -34.78 |
| 195 | 123 | 1.98 | -2582 | -41 |
| 196 | 124 | 2.03 | -2586 | -42.35 |
| 197 | 124 | 1.93 | -2595 | -40.44 |
| 198 | 124 | 2.21 | -2587 | -46.20 |
| 199 | 124 | 1.93 | -2588 | -40.34 |
| 200 | 124 | 1.94 | -2603 | -40.67 |
| 201 | 124 | 1.83 | -2594 | -38.35 |


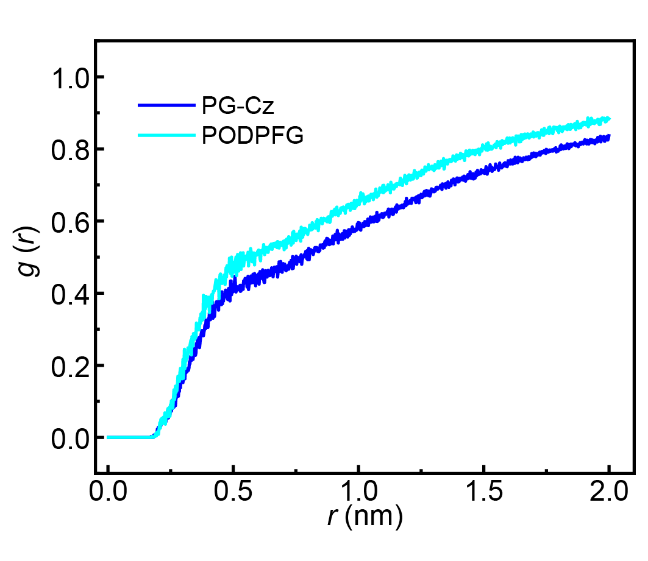


**Figure S1: The radical distributed function (*g* (*r*)) of PG-Cz and PODPFG.**

**Figure S2: The molecular dynamic simulation of stretching PODPFG-based entanglement aggregate.**

**Figure S3: The molecular dynamic simulation of stretching PG-Cz-based entanglement aggregate.**

**
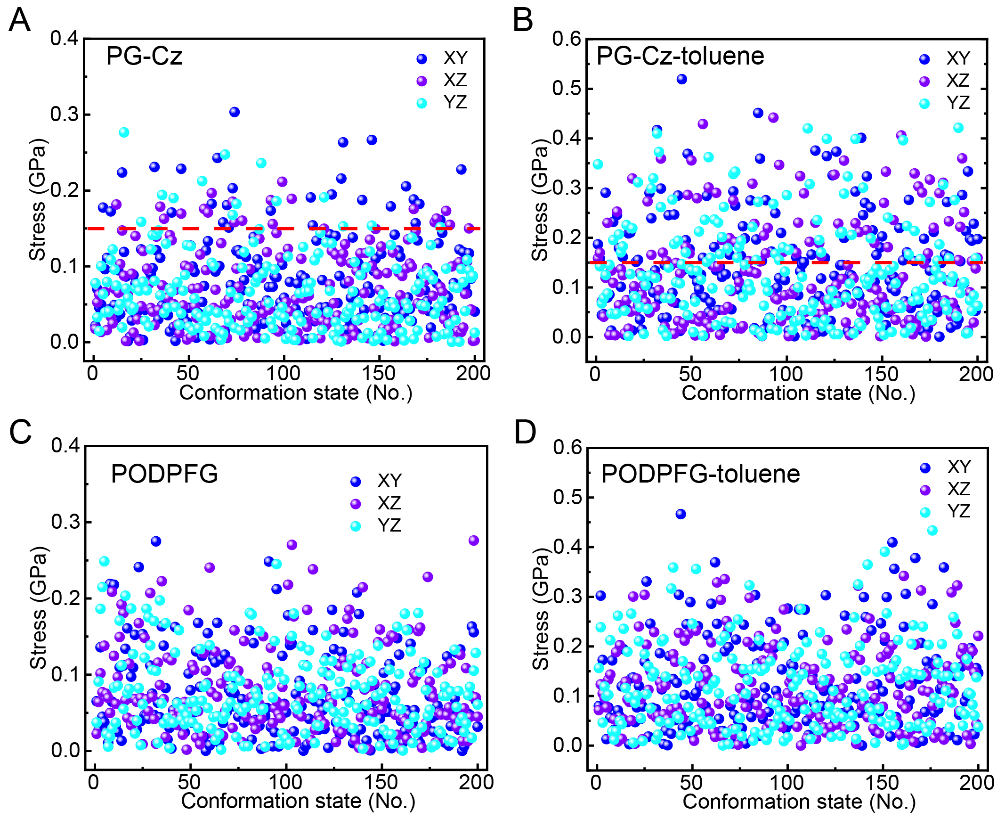
**

**Figure S4: Microscopic stress of nanopolymer-based aggregate systems (in the form of pressure).** The stress is related to the molecular motions and intermolecular interactions in the periodic systems. (A) The PG-Cz aggregate system. (B) The co-existing system of PG-Cz and toluene molecules. (C) The PODPFG aggregate system. (D) The co-existing system of PODPFG and toluene molecules.

Stresses or other microscopic forces, such as in the form of surface tension, are present in the PG-Cz aggregates (due to strong interactions between toluene solvents and PG-Cz chains, along with the motions of toluene solvents). The molecular dynamic simulation is used to roughly evaluate these microscopic stresses (calculated under the NVT ensemble). To avoid the random values in the molecular dynamic simulation, we calculate 200 conformational states of aggregate systems (each with three stress plane directions). In Figure S4A, the PG-Cz aggregate system has dominantly microscopic stresses of 0~0.15 GPa (accounting for 89%), lower than the disentanglement threshold (~0.3 GPa). 11% account of stresses (0.15~0.30 GPa) are approximate to the disentanglement threshold, while none of the stress values exceeds the disentanglement threshold to destroy entanglement systems. However, in Figure S4B, the co-existing system with PG-Cz and toluene molecules exhibits an increase in microscopic stresses, where the stress of 0.15~0.30 GPa (accounting for 27%) and, especially, the stress of 0.3~0.5 GPa (up to or higher than the disentanglement threshold, accounting for 9%) can induce disentanglement behaviors for the generation of crack regions. In this case, the PG-Cz aggregate with a weak entanglement strength cannot bear these microscopic stresses, and thus the partly interchain disentanglement occurs for the generation of crack regions.

As a control system, the molecular dynamic simulation of PODPFG aggregate systems (that cannot form cracks during the same processing) was also performed to calculate the microscopic stresses. These results indicate that almost all of the stresses (in the ranges of 0~0.5 GPa, Figure S4C, and S4D), including with the presence of toluene solvents, are lower than half of the disentanglement threshold (≥1.2 GPa). In this case, PODPFG aggregate has a stronger interchain entanglement strength to bear these microscopic stresses. Thus, the disentanglement behaviors cannot occur in the PODPFG aggregate, and crack regions are not formed easily. These results are also consistent with the experimental observation that a large-scale continuous film is achieved with few crack regions after the evaporation of toluene solvents.

**
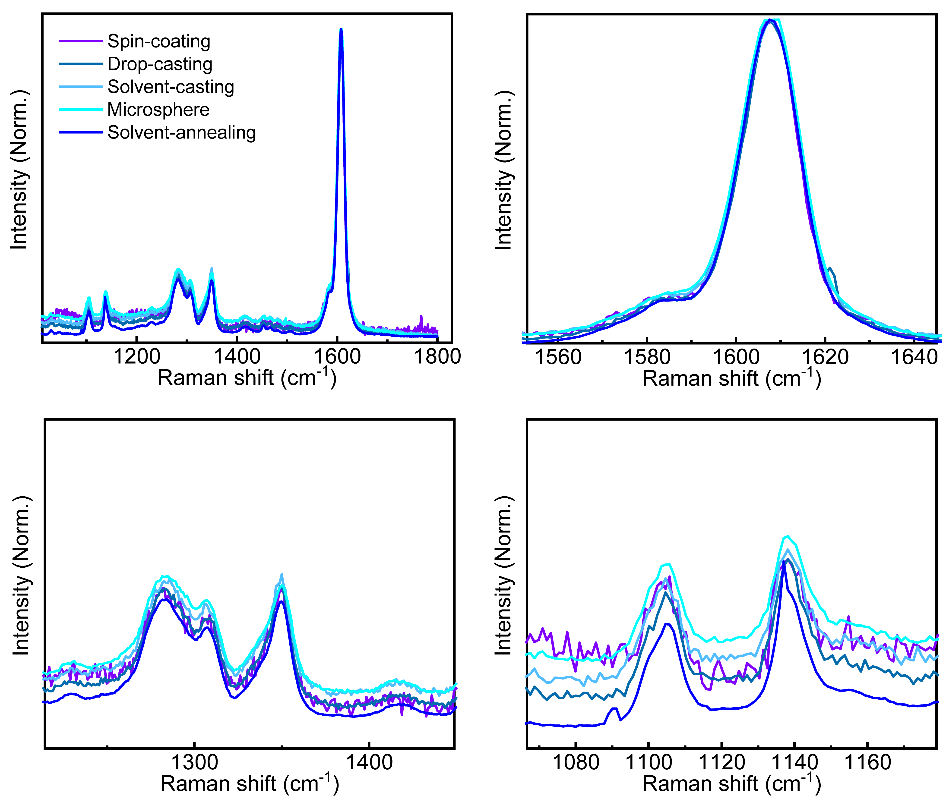
**

**Figure S5: The Raman spectra of PG-Cz-based various films (including the spin-coating, drop-casting, solvent-casting, and solvent-annealing films).** The Raman shifts at 1580~1630 cm^-1^ can be assigned to C=C vibrational stretching on aromatic moieties, where the shoulder peak at 1590 cm^-1^ is related to the conformational planarization (*β*-phase) on polyfluorene backbones [1] with more delocalized π-electronics for π-π conjugation effect and lowering the vibrational energy of C=C bonds. The other intensive Raman peaks at 1608 cm^-1^ can be assigned to the C=C vibrational stretching on fluorenyl moieties with higher dihedral angles between adjacent fluorenyl groups. The Raman shifts at 1283, 1306 and 1350 cm^-1^ are assigned to the stretching vibration of C-C bonds on polyfluorene main-chains, where the more planar conformation results in the red-shift of these peaks, because the stronger π-π conjugation effect enhances the C-C bond strength. Other bands at 1105~1138 cm^-1^ can be assigned to the in-plane bending of C(sp^2^)-H bonds on adjacent fluorenyl groups. The above features firmly reveal that the main-chain conformation of PG-Cz is maintained in various aggregate states (on the spin-coating, drop-casting, solvent-casting, and solvent-annealing films).


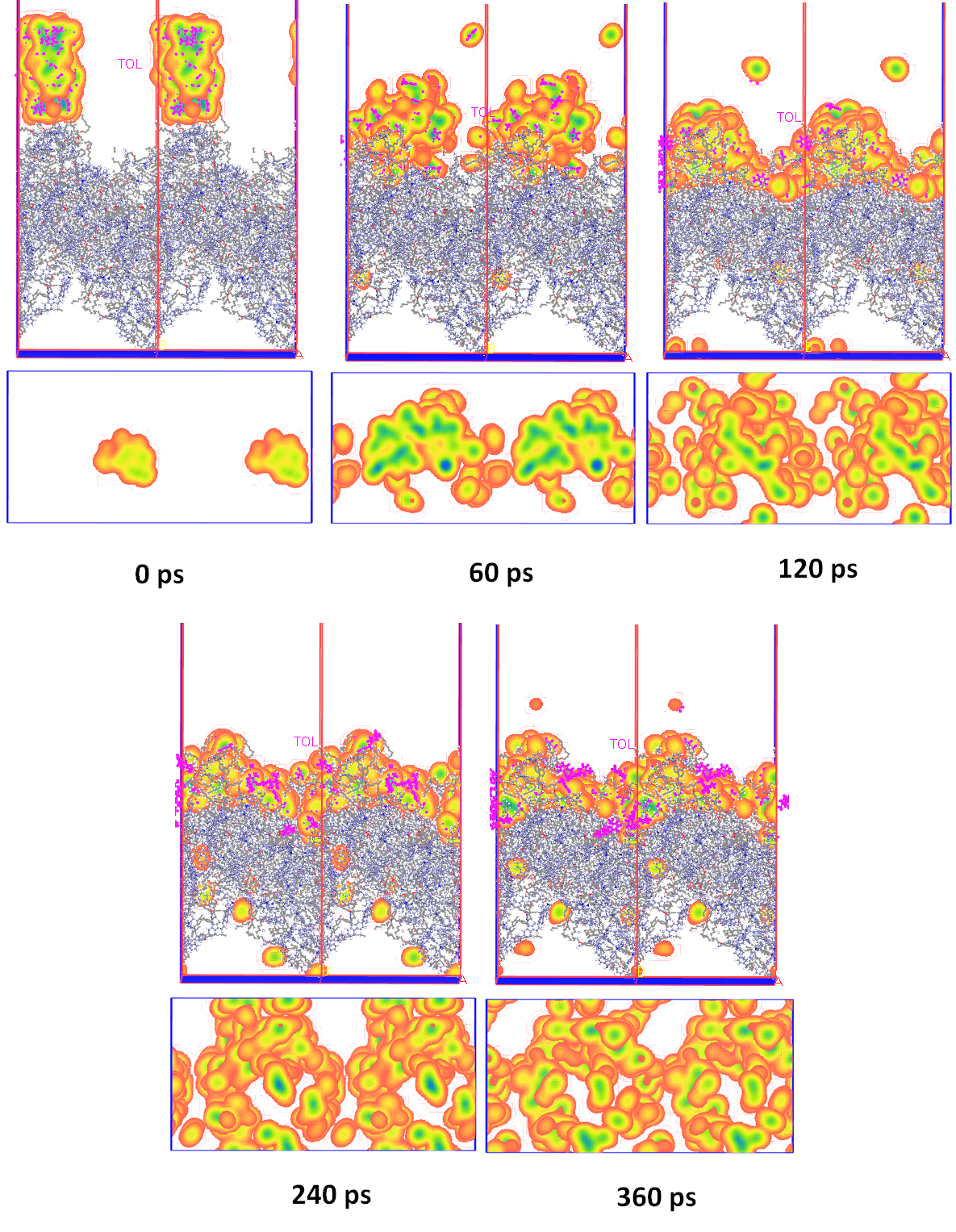


**Figure S6: The molecular dynamic simulation of toluene solvent dropped onto the deposited PG-Cz aggregate surface.** The orange pattern is defined as the density distribution of toluene molecules.

**Figure S7: The molecular dynamic simulation of PG-Cz chains with toluene molecules.**

**Figure S8: The intermolecular interaction energy (*E*_inter_) between PG-Cz chains and toluene solvent m****olecules.** The *E*_inter_ consists of total potential (A and B), noncovalent interactions (C and D), van der Waals (E and F), and electrostatic interactions (G and H).

**Figure S9: The *E*_inter_ values between PG-Cz chains and Toluene.**


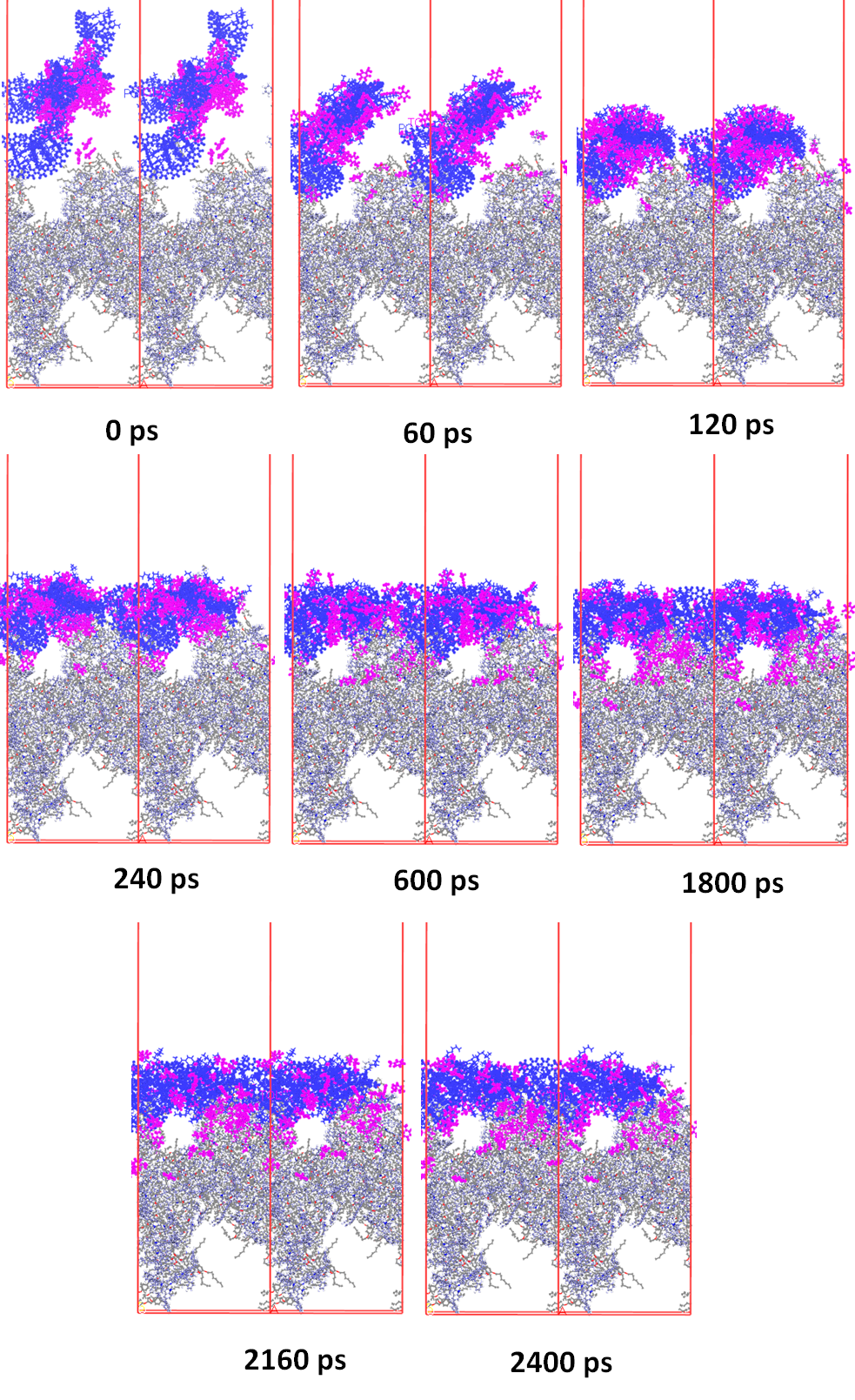


**Figure S10: The molecular dynamic simulation of PG-Cz solution dropped onto the deposited PG-Cz aggregate surface.** The PG-Cz chains are shown in blue, and the toluene molecules are shown in purple.

**Figure S11: The single-chain end-to-end distance (*L*_e_) of PG-Cz chains as the function of time, with toluene or EtOH environment.**

**Figure S12: The interchain distance of PG-Cz chains in a toluene or EtOH environment.**

**Figure S13: The detection of intermolecular interactions between PG-Cz and EtOH solvent.**


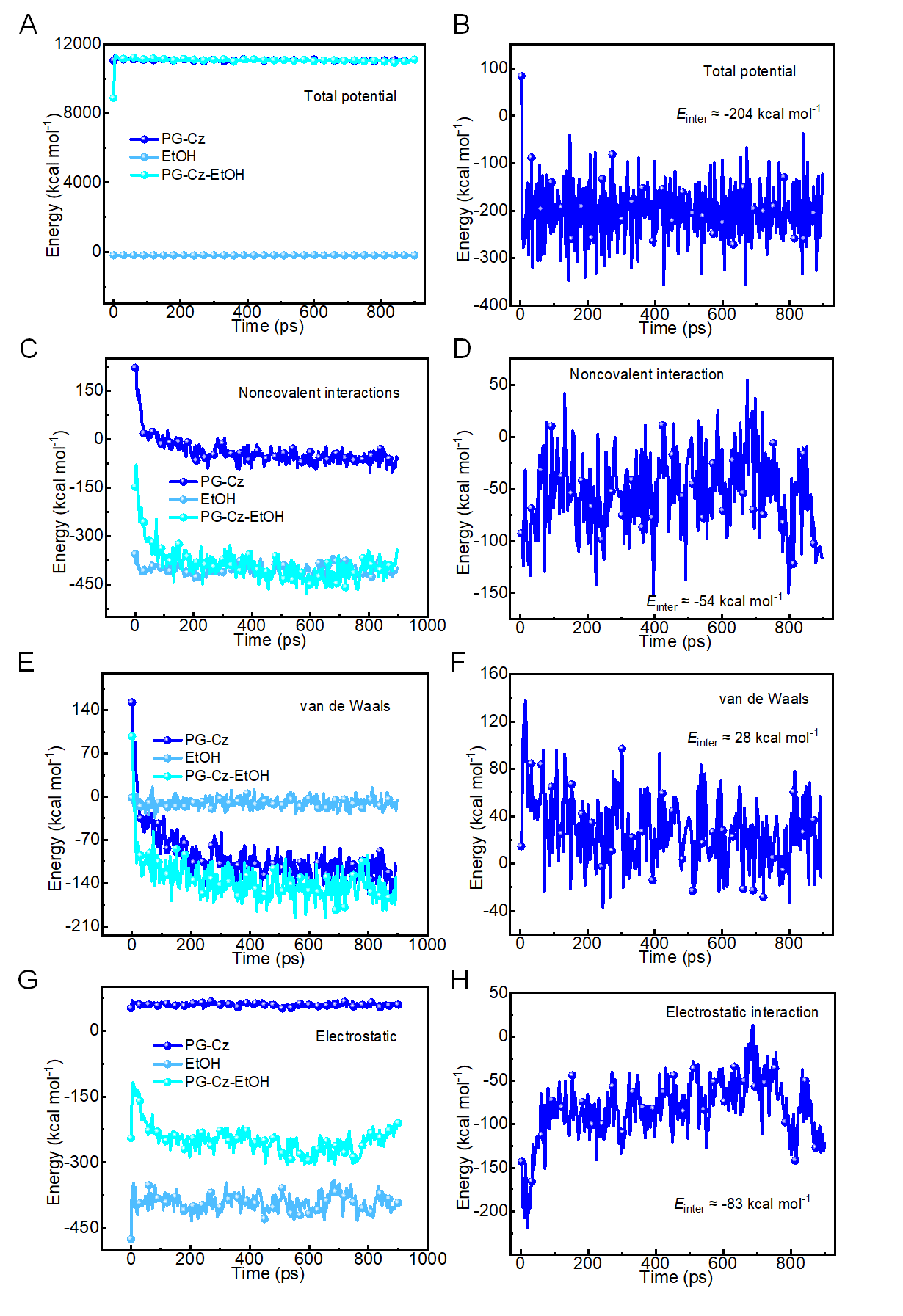


**Figure S14: The intermolecular interaction energy (*E*_inter_) between PG-Cz chains and ethanol solvent molecules.** The *E*_inter_ consists of total potential (A and B), noncovalent interactions (C and D), van de Waals (E and F), and electrostatic interactions (G and H).

**Figure S15: The *E*_inter_ values between PG-Cz chains and EtOH.**


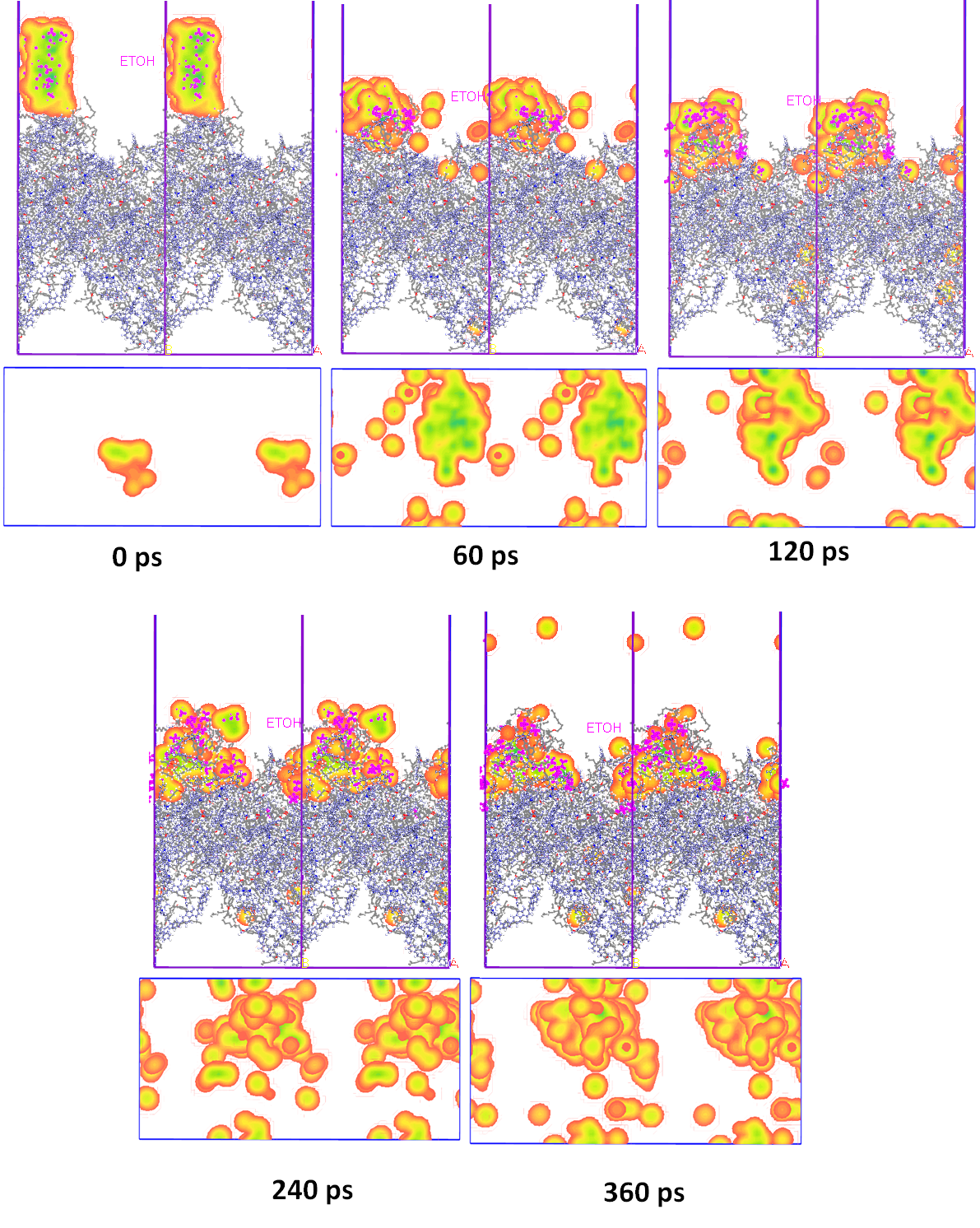


**Figure S16: The molecular dynamic simulation of EtOH solvent dropped onto the deposited PG-Cz aggregate surface.** The orange pattern is defined as the density distribution of ethanol molecules.


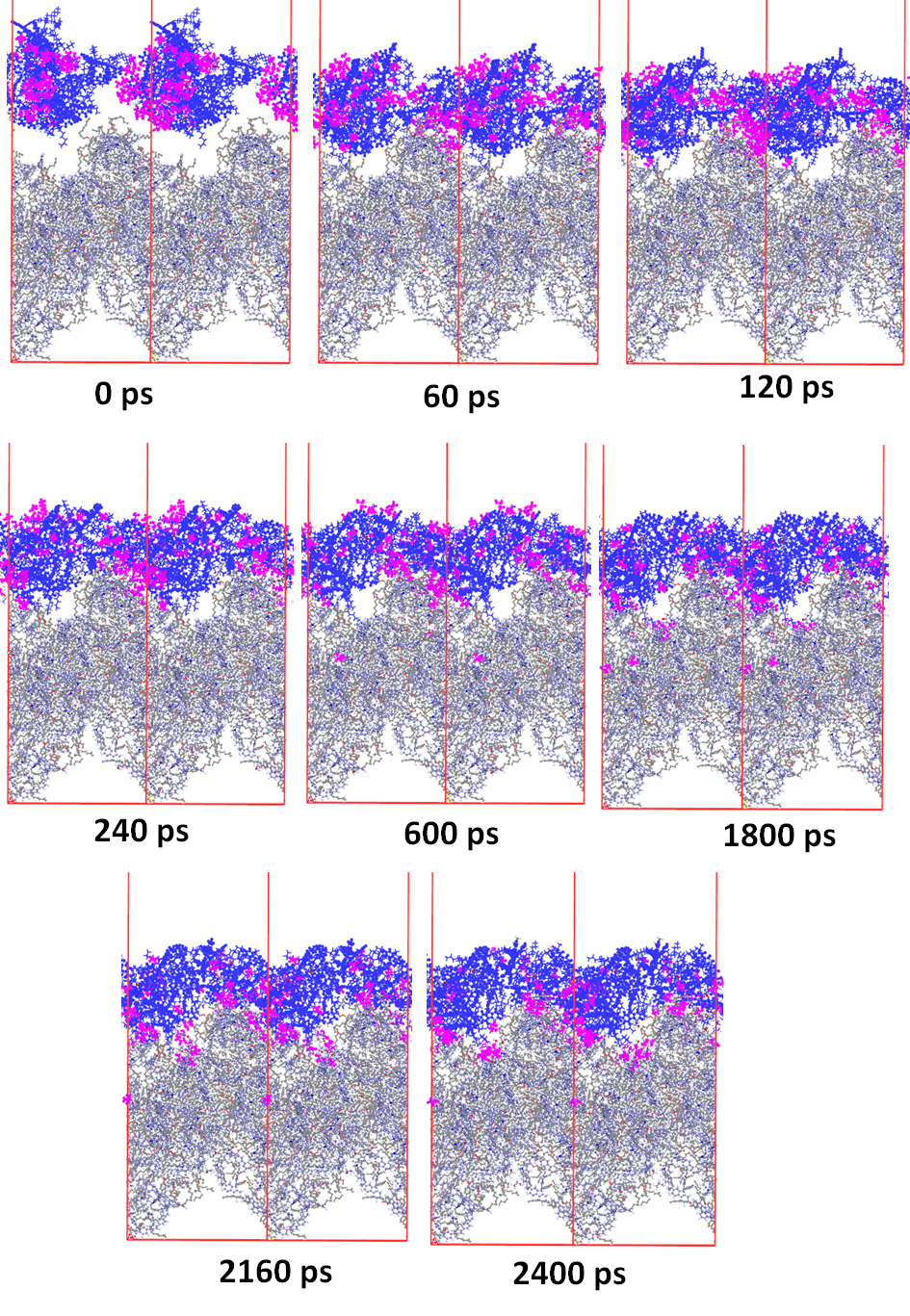


**Figure S17: The molecular dynamic simulation of PG-Cz solution dropped onto the deposited PG-Cz aggregate surface.** The PG-Cz chains are shown in blue, and the toluene molecules are shown in purple.

**Figure S18: The Maldi-ToF-MS of PG-Cz oligomers (in linear mode).** We detected the ionic peaks m/z = 2027, 4053, and 6078 that are consistent with the *DP* = 1, 2, and 3, respectively, where the molecular weight of the PG-Cz monomer is evaluated as 2025. The m/z = 8101 is also detected which is approximate to *DP* = 4 of PG-Cz. The deviation of molecular weight in linear detecting mode cannot be completely avoided. Nevertheless, the above results can confirm the structure of a polygrid consisting of nanogrid repeat units.

**Figure S19. The GPC spectra of PG-Cz oligomers.**

**Table S2: The exact molecular weight (*M*) of PG-Cz oligomers as a variation of elution time (*t*).** It is noted that the assignment of DP is optimized by the higher factor of determination in linear fitting (*R*^2^ = 0.98988).

| *DP* | Elution time (min) | Exact mass (Da) | lg *M* |
| --- | --- | --- | --- |
| 2 | 23.94 | 4052 | 3.61 |
| 3 | 23.15 | 6078 | 3.78 |
| 5 | 21.56 | 10126 | 3.91 |
| 6 | 20.99 | 12150 | 4.01 |
| 7 | 20.57 | 13289 | 4.08 |

The number-average and weight-average molecular weight (denoted as *M*_n_ and *M*_w_, respectively) were calculated according the standard of PG-Cz oligomers (Figure S19, the degree of polymerization *DP* = 2~7) in GPC spectra, in terms of our previous work [2]. The linear calibration equation is fitted as **ES3**: lg *M* = -0.156 *t* + 7.367. The higher molecular weight values of PG-Cz chains were assigned and calculated through extrapolating its calibration equation, as listed in the Table S3. The *M*_n_ and *M*_w_ values are evaluated based on the Equation **ES4** and **ES5**, respectively (considering $\int_{o}^{\infty} P\left( M \right)dM=1$, where *P*(M) can be denoted as the percentage of the polymer chain with specific MW or degree of polymerization (*DP*)).

$M_{n}$ $=$ $\frac{\sum N_{i}M_{i}}{\sum N_{i}}= \int_{0}^{\infty} p\left( M \right)MdM$ **ES4**

$M_{w}$ = $\frac{\sum N_{i}M_{i}^{2}}{\sum N_{i}M_{i}}= \frac{\int_{0}^{\infty} p\left( M \right)M^{2}dM}{M_{n}}$ **ES5**

**Table S3: The elution times of PG-Cz chains with specific *DP*.**

| *DP* | Elution time (min) | Exact mass (Da) | lg *M* |
| --- | --- | --- | --- |
| 8  9  10  11  12  13  14  15  16  17 | 20.24  19.91  19.62  19.35  19.11  18.89  18.68  18.49  18.31  18.14 | 16198  18222  20246  22270  24294  26318  28342  30366  32390  34414 | 4.21  4.26  4.31  4.35  4.39  4.42  4.45  4.48  4.51  4.54 |
| 18 | 17.98 | 36438 | 4.56 |

**Figure S20: The GPC spectra of PG-Cz.** (A) Raw GPC spectra. (B) Detailed analysis. The total signal peak was afforded through the fitting of gauss peaks with individually pure *DP* values. The height of red stars represents the percentage of specific *DP*. *DP*_n_ and *DP*_w_ were calculated according to the integration of each *DP* percentage.

**Self-assembled morphologies**

**Figure S21: The SAED pattern of PG-Cz-based multilayer crack from the solvent-casting method.**

**Figure S22: The XRD of PG-Cz-based multilayer crack from the solvent-casting method.**


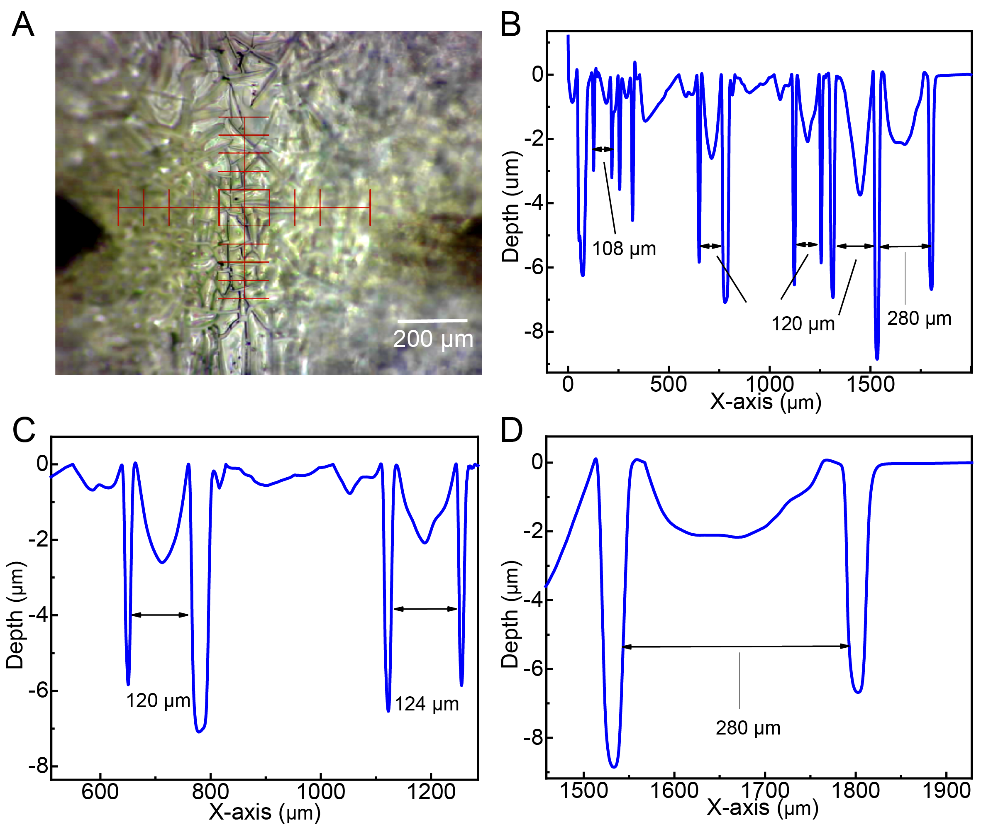


**Figure S23: The surface morphologies of PG-Cz-based interface cracks from the drop-casting method.** (A) The Surface images observed by profile characterizations. The red lines are scale bars with a total length of 2200 μm. The height profiles can be extracted and are provided in (B) (C) (D).

**
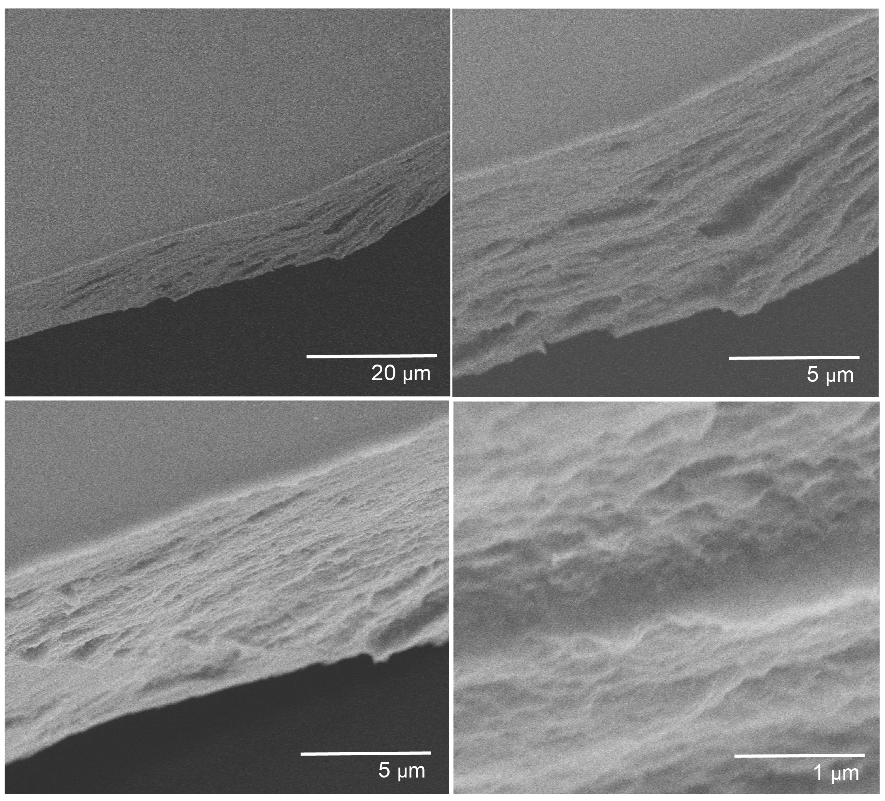
**

**Figure S24: The SEM images of multilayer structure from the drop-casting method.**


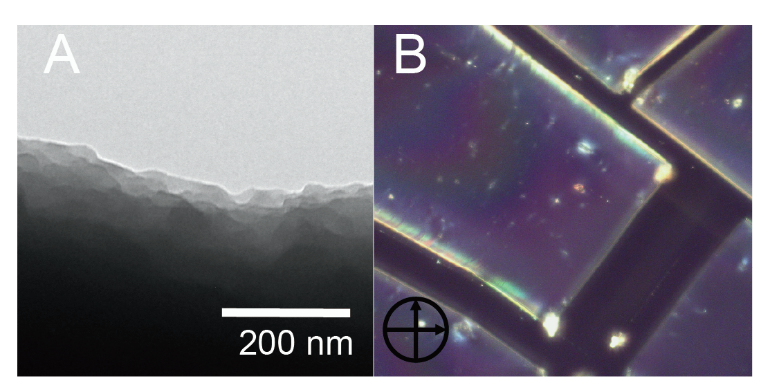


**Figure S25: The TEM (A) and POM (B) images of multilayer cracks from the drop-casting method.**


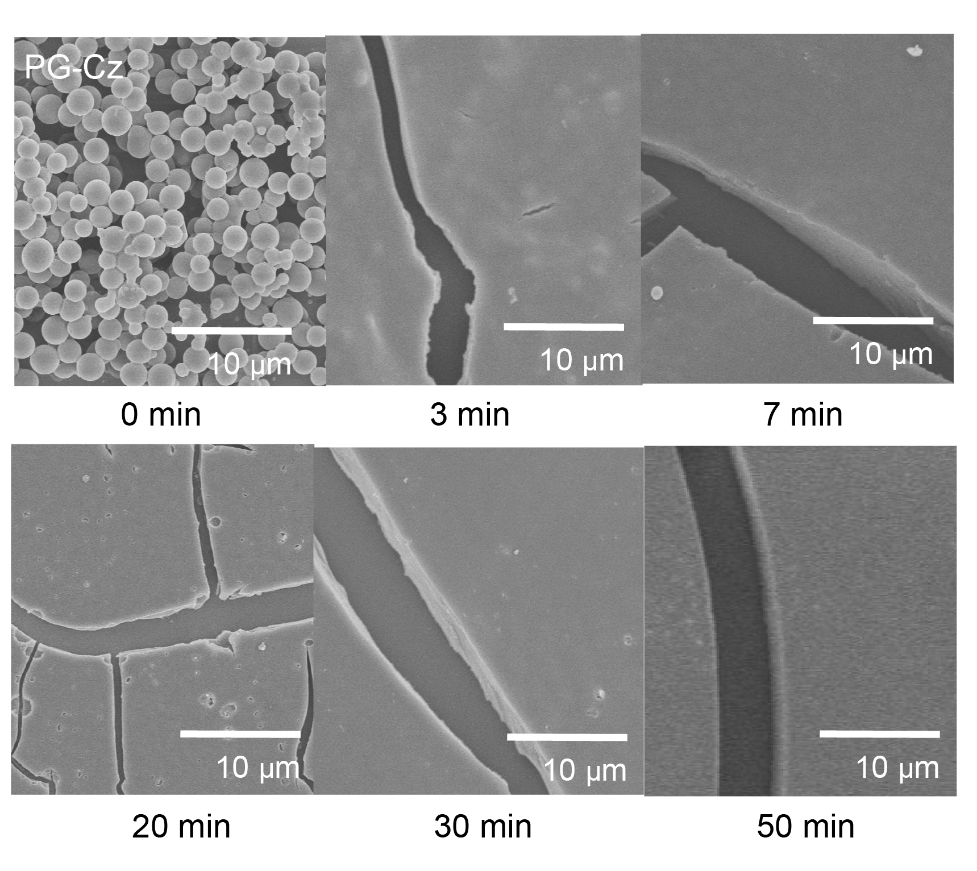


**Figure S26: The morphological transformation of PG-Cz from microsphere films to multilayer crack morphologies through the solvent-annealing method.**


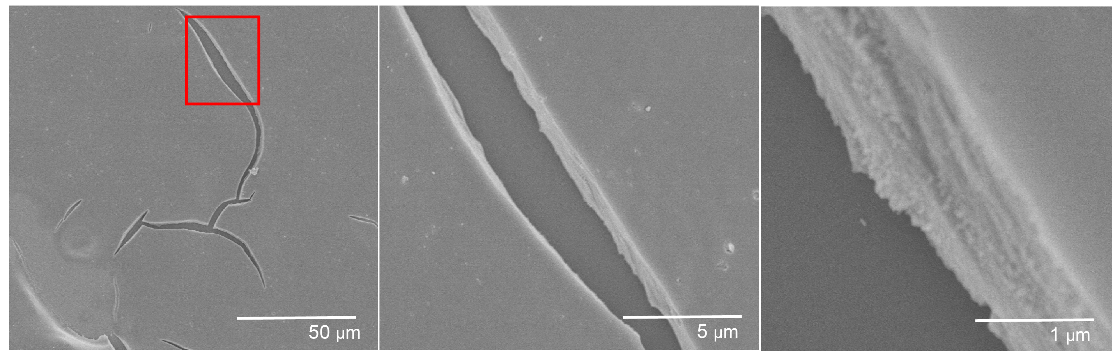


**Figure S27: The deep observation of Morphological transformation of PG-Cz chains at 30 min.** The red regions (a, d, and g) are magnified.


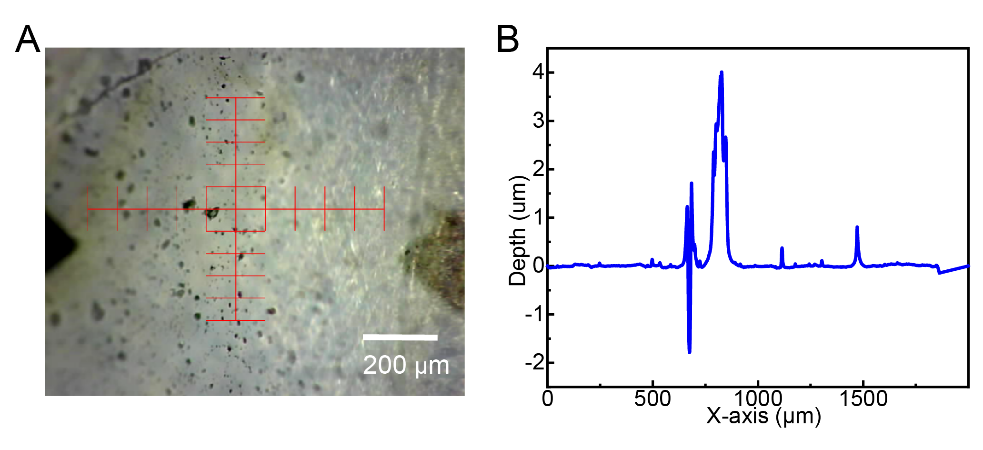


**Figure S28: The surface morphologies from solvent-annealing.** The number of interface cracks is diminished.

**Figure S29: The HR-TEM images of PG-Cz-based solvent-annealing film (A-E).** The SAED pattern (F) is also provided.

**Demonstrations of optical features**

**
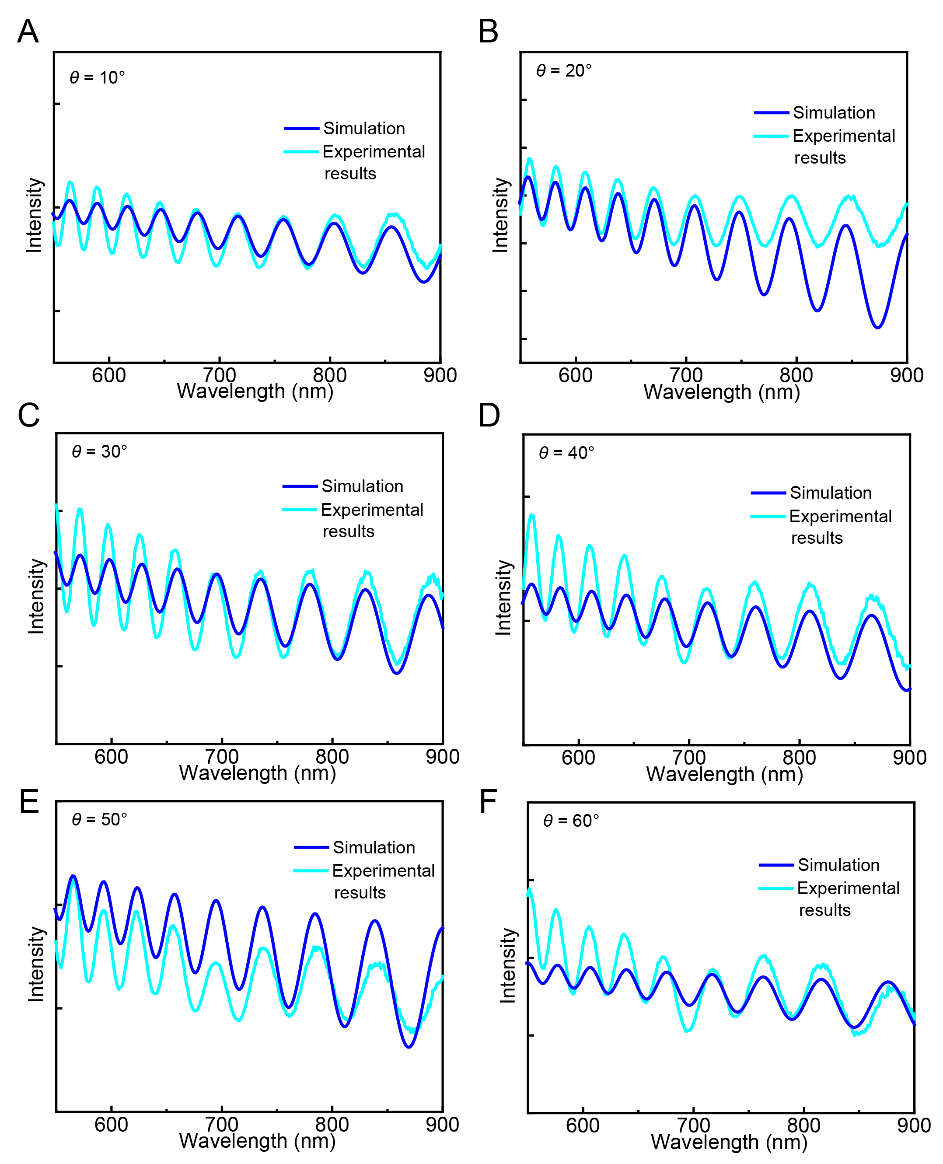
**

**Figure S30: The simulation of reflected interference fringe in the incident angle range of 10~60°.** The absolute intensity feature, linked to the spectra intensity, is neglected in this part.

**
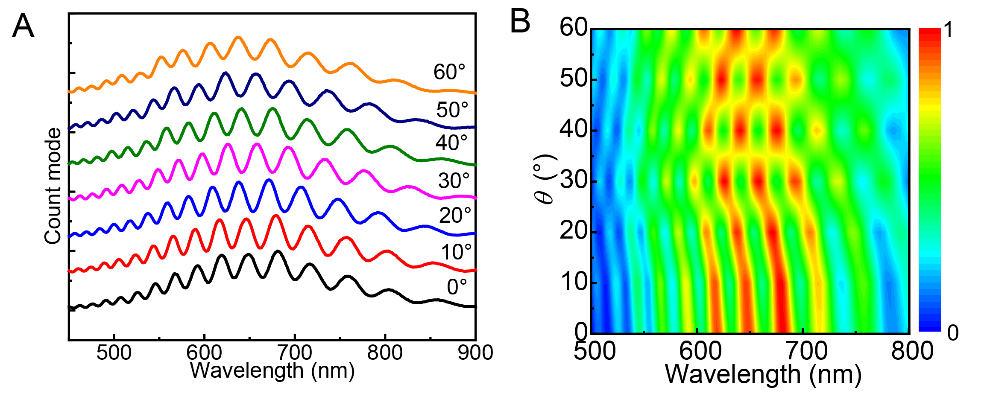
**

**Figure S31: The count mode spectra of PG-Cz-based solvent-annealing film.** (A) One-dimensional spectra. (B) Two-dimensional mapping image.

**
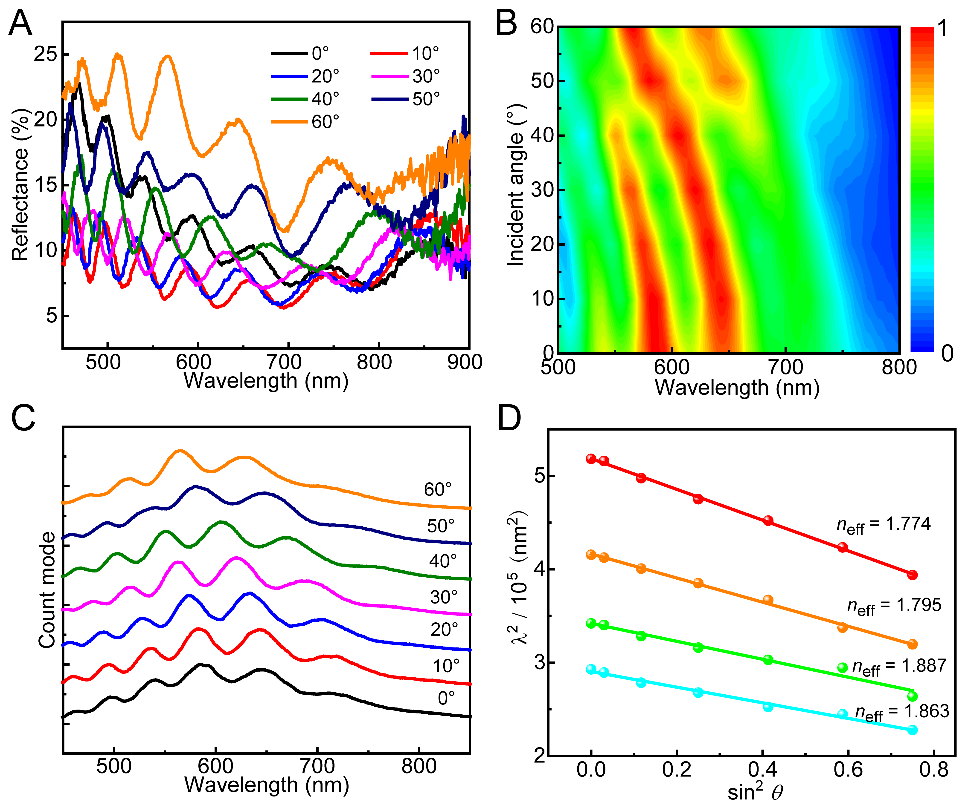
**

**Figure S32: The angle-dependent reflectance spectra of PG-Cz-based solvent-casting film.** (A) The wavelength-dependent absolute reflectance in the range of 500~800 nm wavelength and 0~60° incident angle. (B) The two-dimensional count mode mapping. (C) The one-dimensional count mode in the incident angle ranges of 0~60°. (D) The relationship between the wavelength peak (denoted as *λ*) and the incident angle. We observed a series of constructive interference fringe in angle-resolved reflectance spectra, which also possesses the angle-dependent blue-shift with increasing incident angles and follows the Bragg-Snell diffraction law. Thus, such PG-Cz-based solvent-casting film with multilayer microstructures is also demonstrated to be one-dimensional photonic crystals. Even so, the addition of material/air interfaces in the crack regions can also result in light scattering that has an adverse influence on the constructive interference fringe.


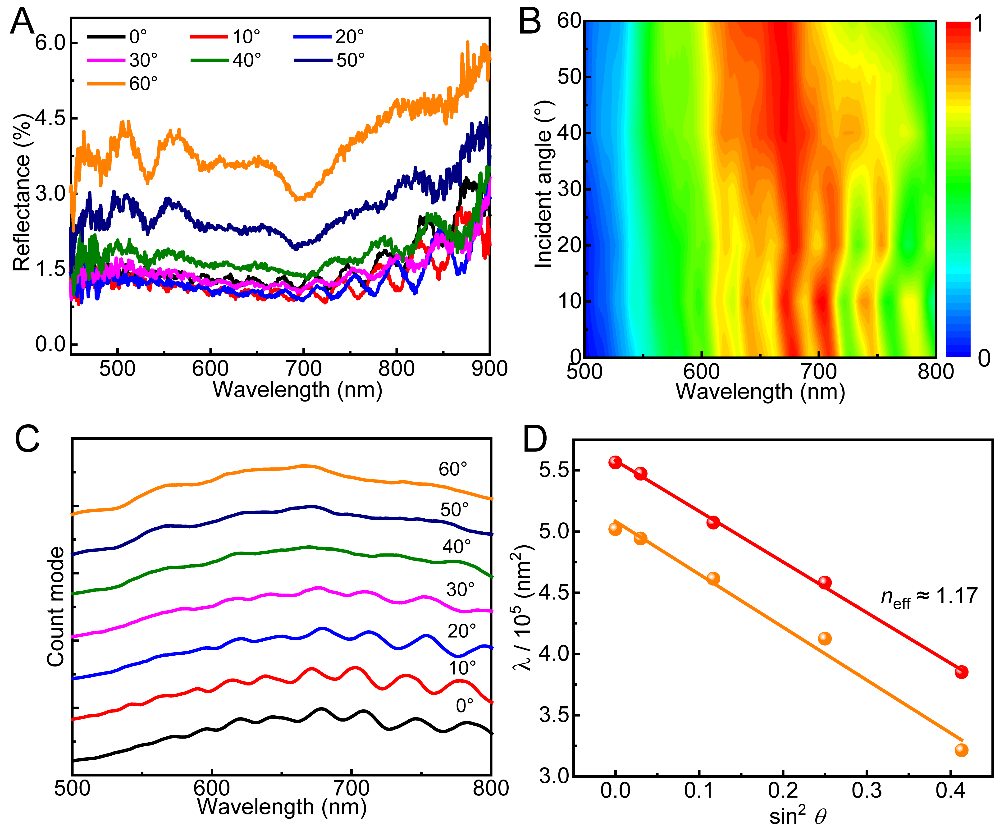


**Figure S33: The angle-dependent reflectance spectra of PG-Cz-based drop-casting film.** (A) The wavelength-dependent absolute reflectance in the range of 500~800 nm wavelength and 0~60° incident angle. (B) The two-dimensional count mode mapping. (C) The one-dimensional count mode in the incident angle ranges of 0~60°. (D) The relationship between the wavelength peak and the incident angle. For a such drop-casting film with interface cracks, a series of constructive interference fringe in angle-resolved reflectance spectra can also be observed if in the small incident angle *θ* = 0~30°, while the larger incident angles seemingly do not show the ordered optical interference. Further, such constructive interference fringe at the small *θ* = 0~40° also exhibits the angle-dependent blue-shift with obeying the Bragg-Snell diffraction law. As a result, such PG-Cz-based drop-casting films also can be identified as one-dimensional photonic crystals with mesoscale periodicity. However, due to more material/air interfaces on more discrete films, the light scattering can be further enhanced. Meanwhile, the more material/air interfaces also increase the air region which decreases the effective refractive index (*n*_eff_) to ~1.17.

The *n*_eff_ values within specific wavelength regions can be extracted from the fitting of the Bragg-Snell diffraction law:

*λ*^2^ = -*k* sin2 *θ* + *b*  **ES6**

Where *k* and *b* are the slope and the intercept of such function. Considering the Bragg-Snell diffraction law:

*mλ* = 2*d* (*n*_eff_^2^ – sin^2^ *θ*)^0.5^ **ES7**

Where *m* and *d* are the optical diffraction order and the interspacing distance, respectively, the *n*_eff_ value can be evaluated based on the equation:

*n*_eff_ = (*b* / *k*)^0.5^  **ES8**

According to the equation **ES8**, the larger *n*_eff_ values correspond to the higher *k* values, enabling more blue-shifted reflectance spectra when increasing the same incident angle.

**
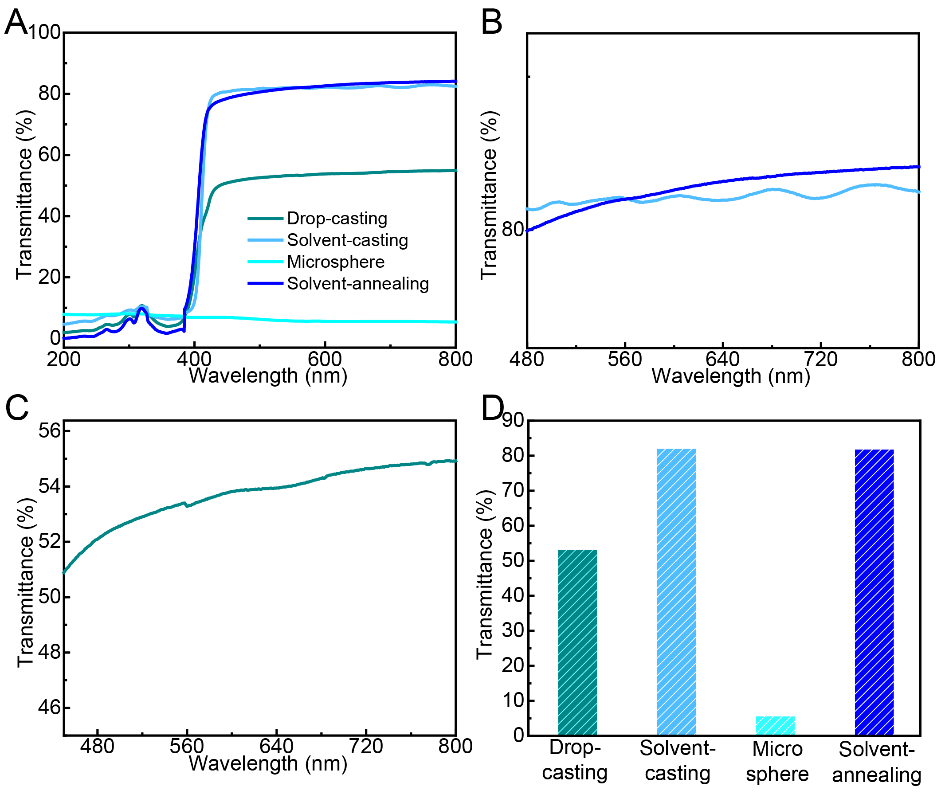
**

**Figure S34: The transmittance of PG-Cz-based various self-assembled films (including drop-casting, solvent-casting, microsphere, and solvent-annealing films).** (A) The transmittance spectra (transformed from the UV absorption) of PG-Cz-based drop-casting, solvent-casting, microsphere, and solvent-annealing films in the wavelength ranges of 200~800 nm. (B) Magnification of the transmittance spectra of PG-Cz-based solvent-casting and solvent-annealing film in the ranges of 480~800 nm wavelength and 70~90% transmittance. (C) Magnification of the transmittance spectra of PG-Cz-based drop-casting film in the ranges of 480~800 nm wavelength and 45~57% transmittance. The transmittance values (at 550 nm for average level) are exhibited in (D).

**Demonstrations on the enhancement of quantum yields**

**
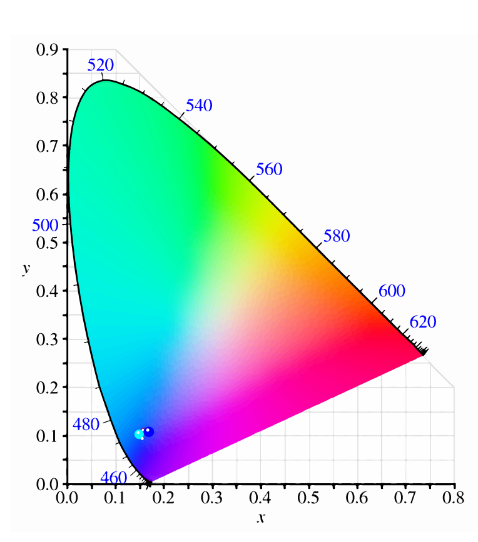
**

**Figure S35: The CIE coordination of PG-Cz-based various films with multilayer crack morphologies (1~10 μm thickness).**

**
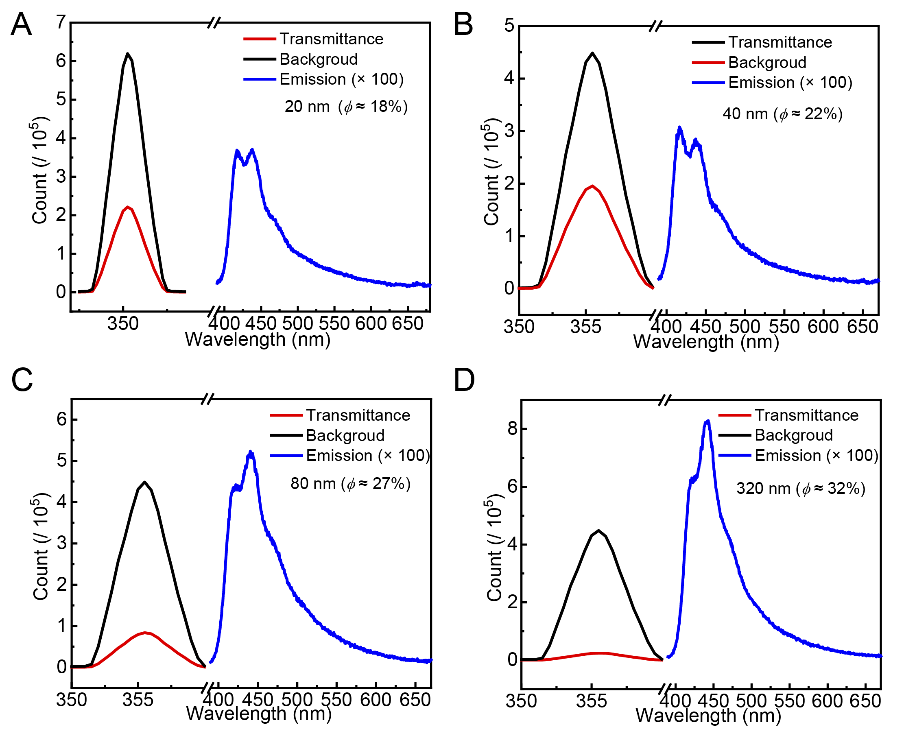
**

**Figure S36: The measurements for the calculation of absolute fluorescence quantum yields on PG-Cz-based spin-coating films with varying thicknesses (20 nm, 40 nm, 80 nm, and 320 nm).** The photonic count (reflected, transmitted, and emitted) is collected by an integral sphere. The thickness of the spin-coating film is controlled by the solution concentration of 5 mg ml^-1^, 10 mg ml^-1^, and 30 mg ml^-1^, respectively, all of which were spin-coated under the same spin-coating rate of 1000 rad s^-1^.


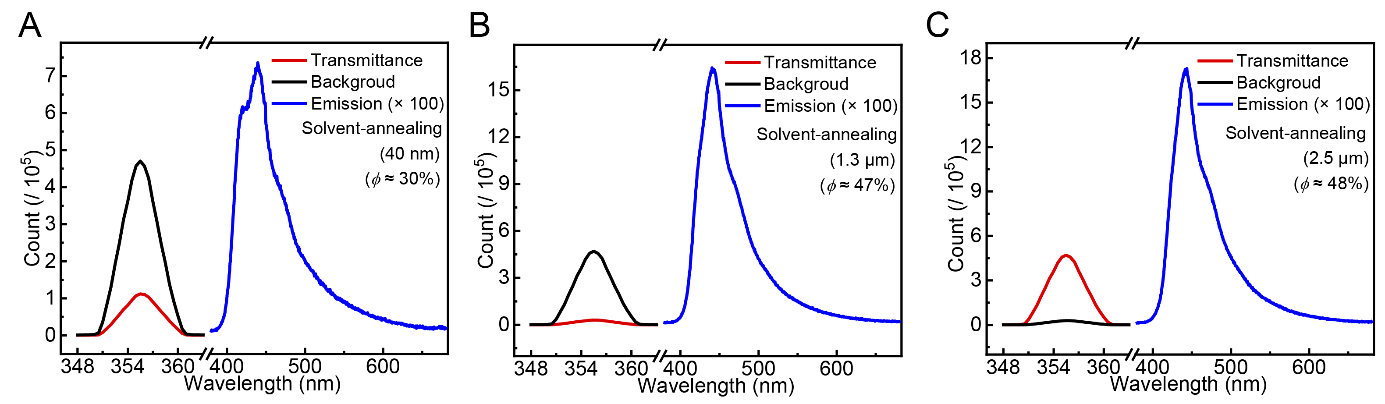


**Figure S37: The measurements for the calculation of absolute fluorescence quantum yields on PG-Cz-based solvent-annealing films with varying thicknesses (40 nm, 1.3 μm, and 2.5 μm).** The photonic count (reflected, transmitted, and emitted) is collected by an integral sphere.


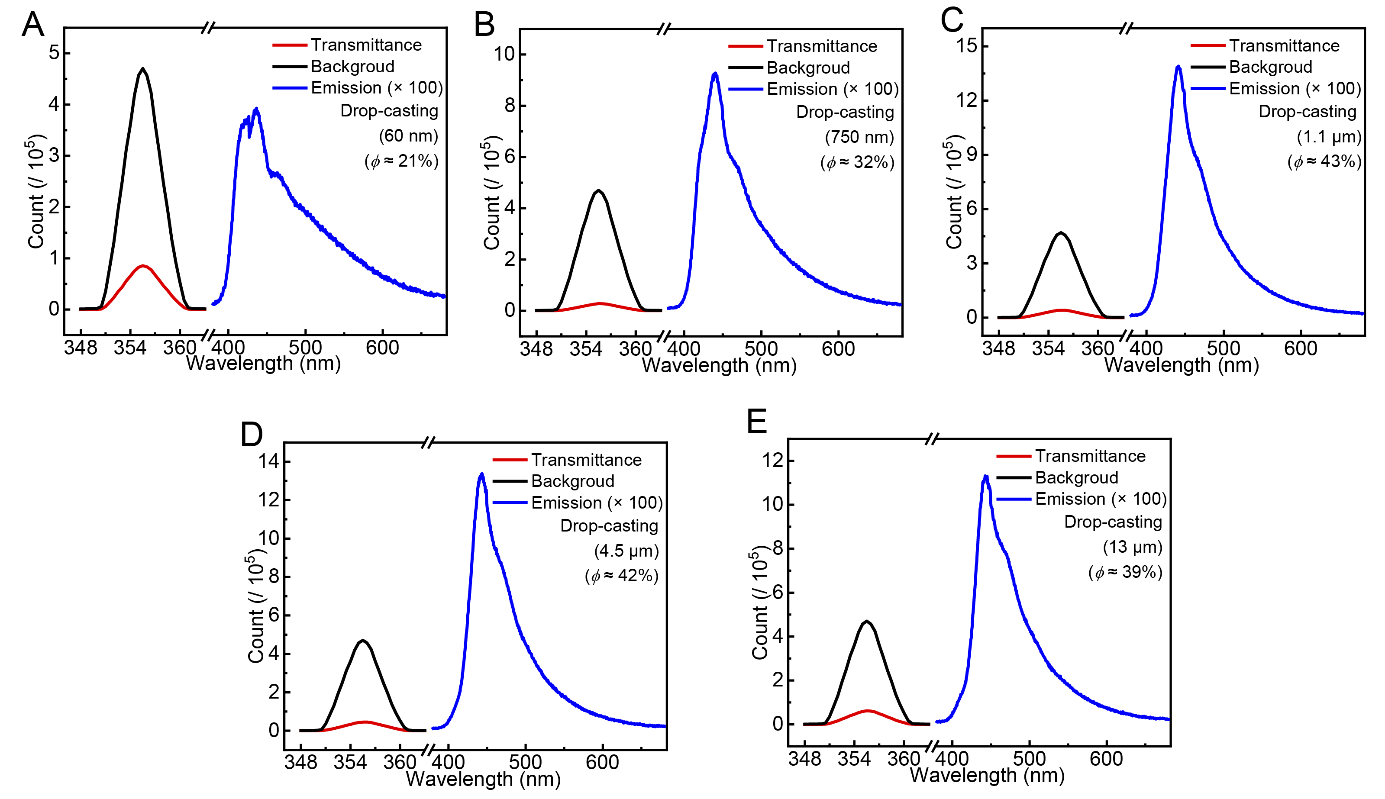


**Figure S38: The measurements for the calculation of absolute fluorescence quantum yields on PG-Cz-based drop-casting films with varying thicknesses (60 nm, 750 nm, 1.1 μm, 4.5 μm, and 13 μm).** The photonic count (reflected, transmitted, and emitted) is collected by an integral sphere.


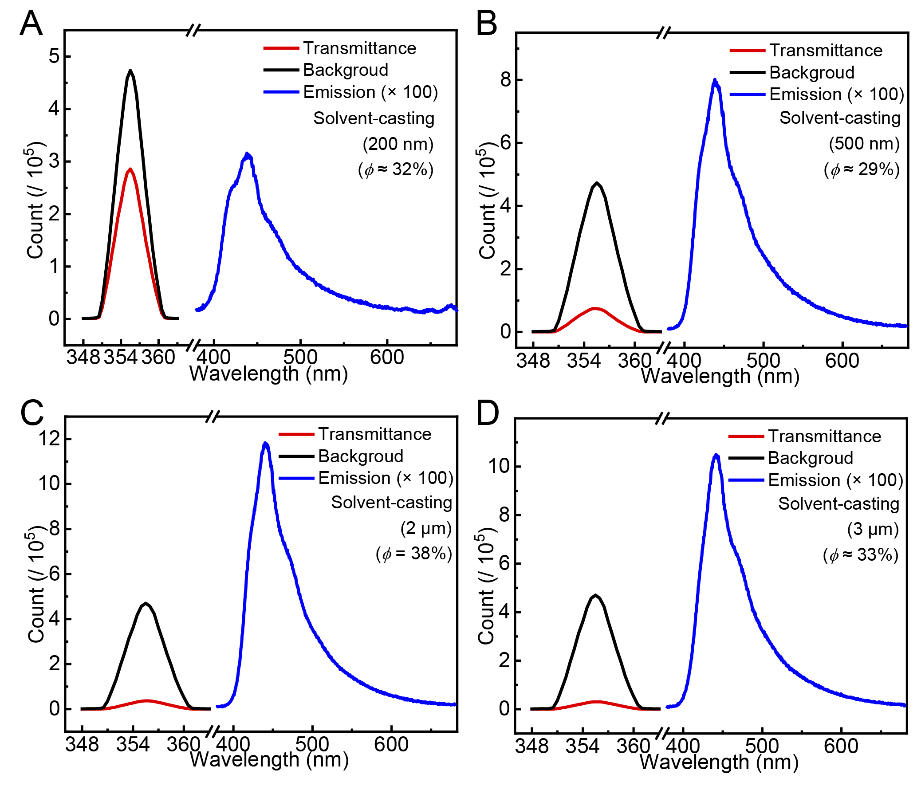


**Figure S39: The measurements for the calculation of absolute fluorescence quantum yields on PG-Cz-based solvent-casting films with varying thicknesses (200 nm, 500 nm, 2 μm, and 3 μm).** The photonic count (reflected, transmitted, and emitted) is collected by an integral sphere.


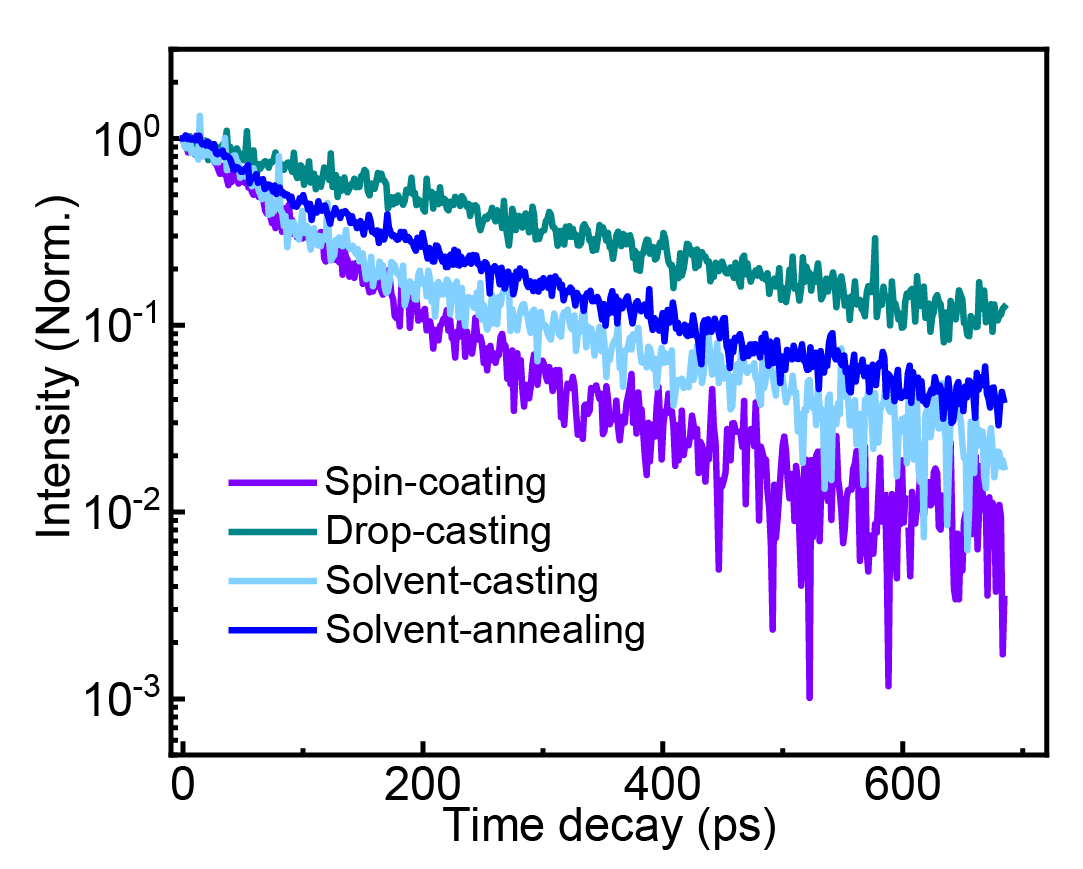


**Figure S40: The fluorescence lifetimes of various films.**


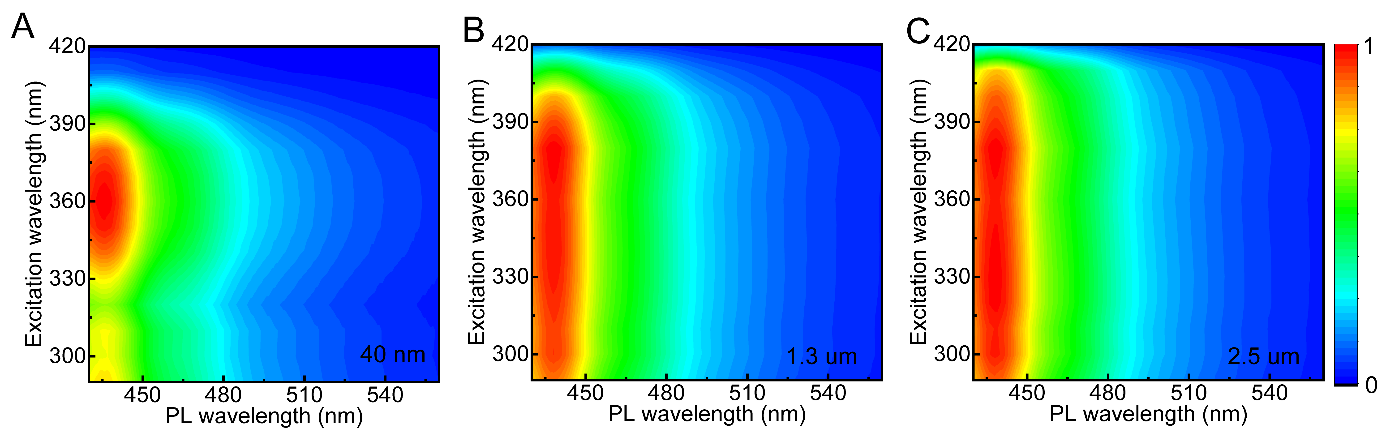


**Figure S41: The excitation spectra of PG-Cz-based solvent-annealing films with various thicknesses.**


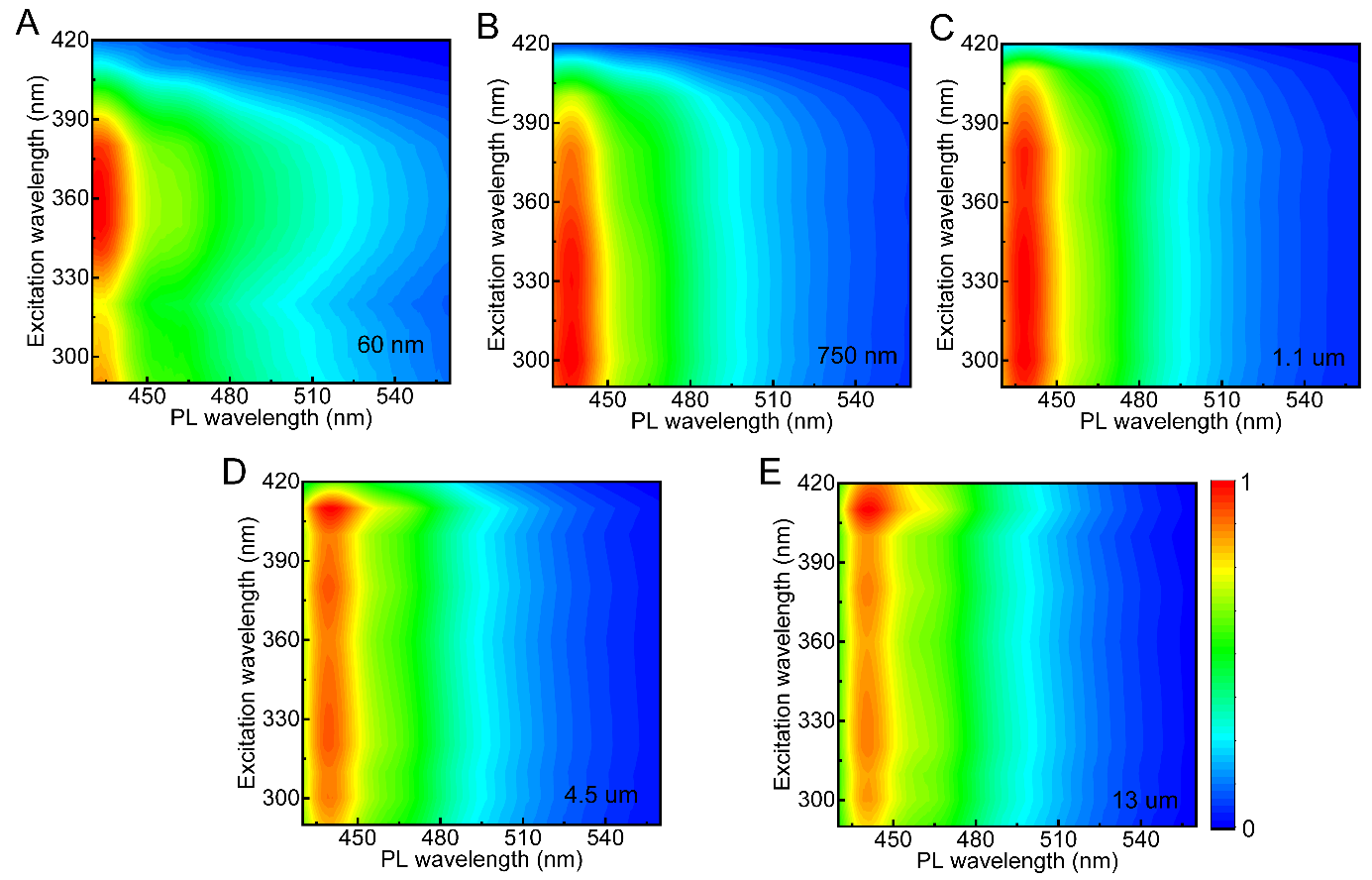


**Figure S42: The excitation spectra of PG-Cz-based drop-casting films with various thicknesses.**


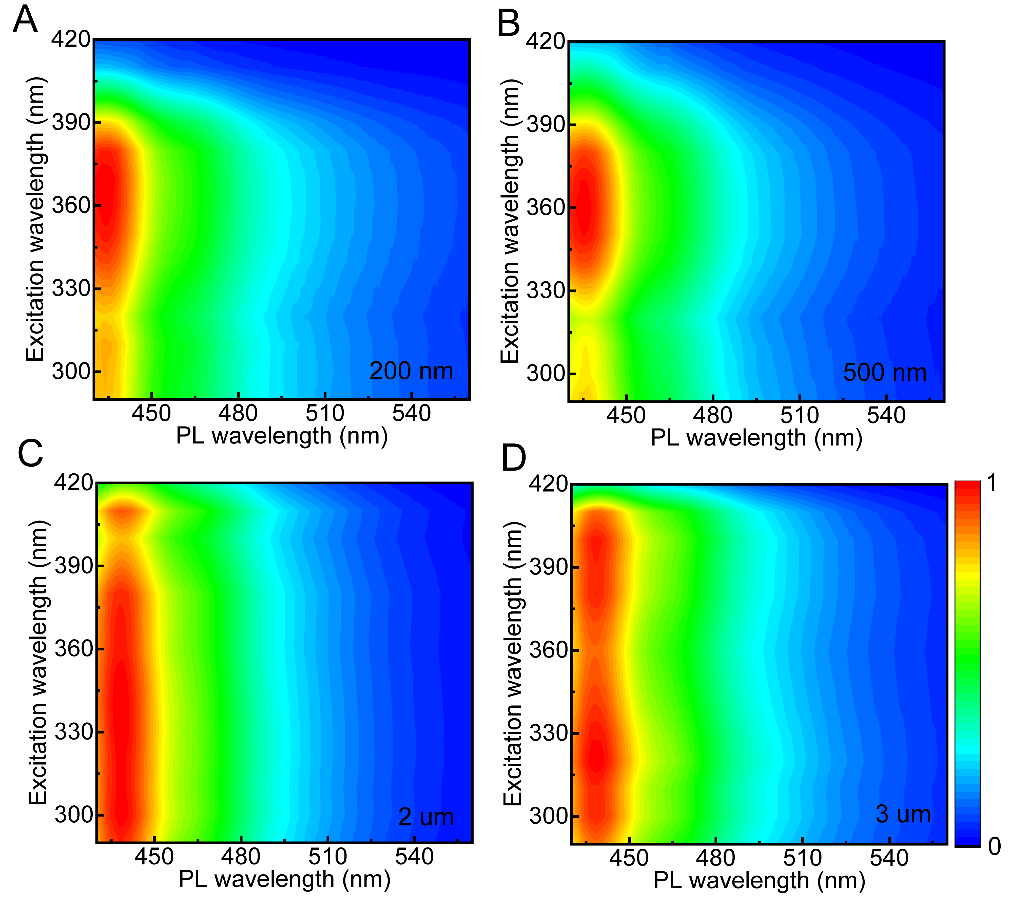


**Figure S43: The excitation spectra of PG-Cz-based solvent-casting films with various thicknesses.**


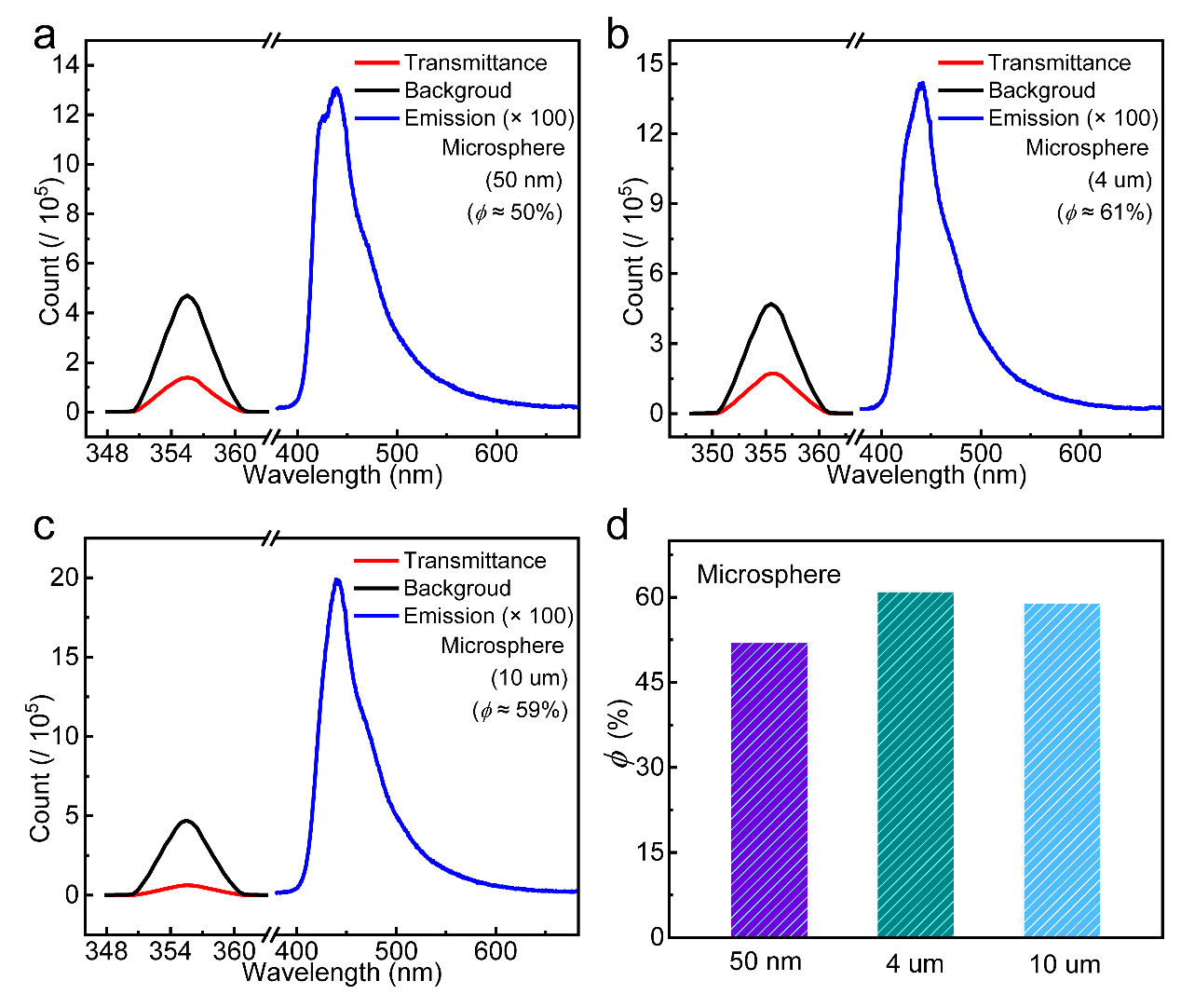


**Figure S44: The measurements for the calculation of absolute fluorescence quantum yields on PG-Cz-based microsphere films with varying thicknesses.**

**
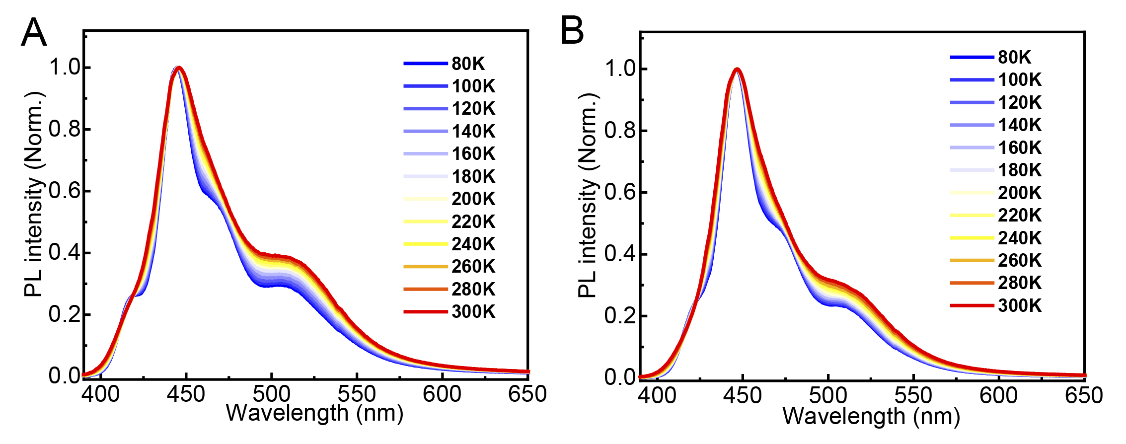
**

**Figure S45: The temperature-dependent PL spectra of PG-Cz-based solvent-casting film (A) and solvent-annealing film (B), under the temperature range of 80~300 K.**

**Figure S46: The temperature-dependent PL spectra of microsphere films.** The intensity ratio of 0-0 to 0-1 emission (*I*_0-0_/*I*_0-1_) is shown in (B).


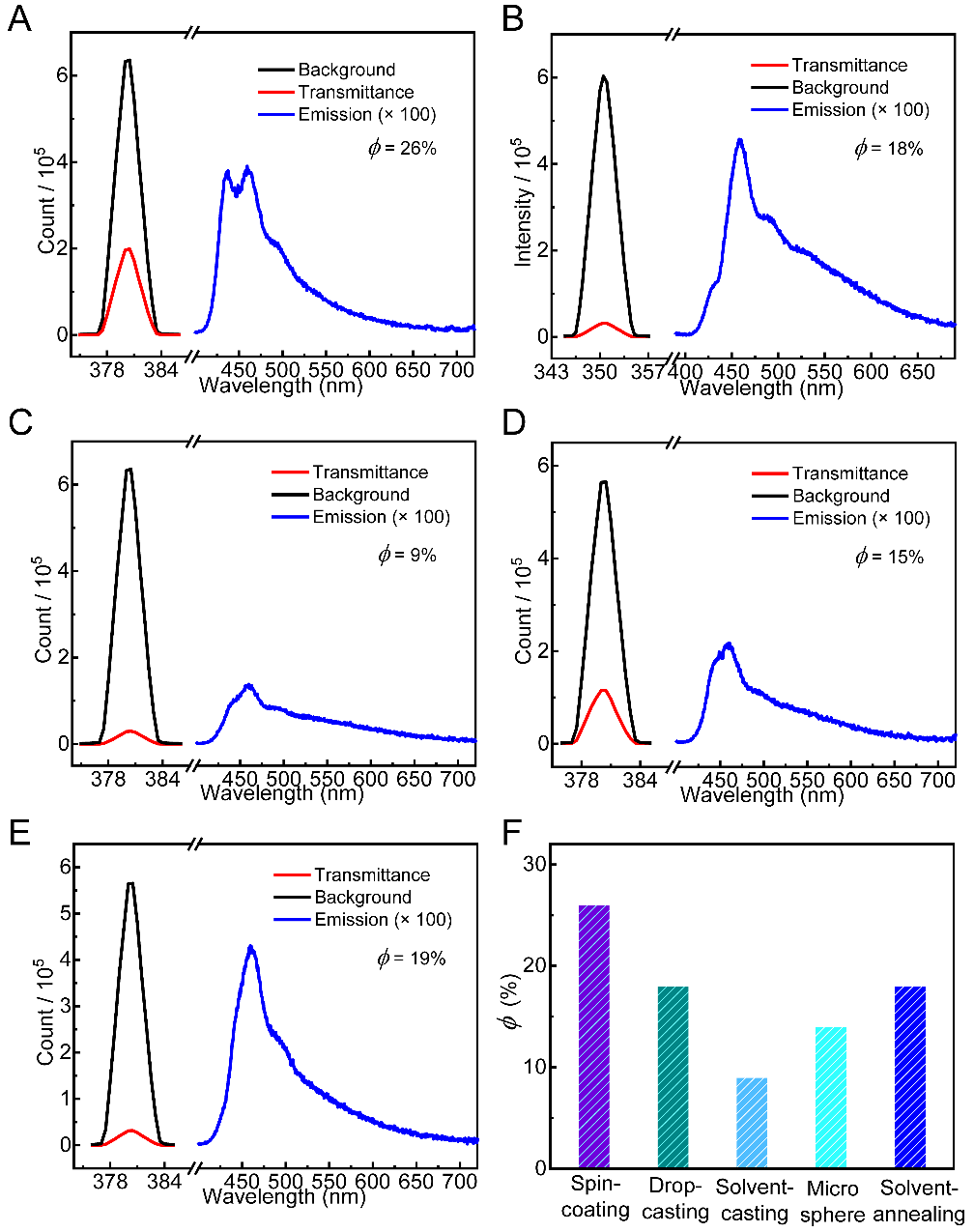


**Figure S47: The measurements for the calculation of absolute fluorescence quantum yields on PODPFG-based various films.**

In general, the enhancement of quantum yields is likely derived from the intrinsic properties (related to the single-chain conformation and the interchain aggregation) or the local electrical field intensity from microstructures (like the Purcell effect or other microcavity effects) [3, 4]. In this work, these multilayer microstructures have micrometer-scale large mode volumes, which should have a negligibly weak Purcell effect (different from the nanoscale mode volume for effectively enhancing spontaneous emission rate) [3]. Thus, the effect of multilayer microstructures (like the cavity effect) on the electromagnetic environment is ruled out for the enhancement of quantum yields. Meanwhile, it is noted that the conjugated polygrid PODPFG has similar multilayer film structures, but both the solvent-casting film and the solvent-annealing film exhibit the diminishment of quantum yields versus that of thin film (Figure S47). These results confirm the negligible influence of multilayer structures (electromagnetic environment) on the enhancement of quantum yields.

We hypothesized that if the multilayer structures make a significant contribution to the enhancement of quantum yields, the measured quantum yields of microsphere structures should be lower. In Figure S44, the microsphere films with 50 nm thickness can display a high quantum yield of 50%, which can be slightly enhanced to 61% under the microsphere thickness of 4 μm. These results indicate that the multilayer structure is not a significant contribution to the enhancement of quantum yields, or the enhancement of quantum yields is not directly related to the microstructures. Deeply, in the microsphere film fabricated from the poor-solvent-induced self-assembly method, the interchain aggregation intensity of PG-Cz should be stronger than that in multilayer films, because the poor solvent favors interchain aggregation and packing through stronger monomer-monomer interactions (confirmed by the molecular dynamic simulation in Figure S14, S15, and S17). These results indicate that the more intense interchain aggregation can cause higher quantum yields of deep-blue emission (even under the microsphere film thickness of 50 nm).

In terms of the interchain aggregation, we compared the above results with the conjugated polygrid PODPFG (previously reported), where the micrometer-thick multilayer stacking films have lower quantum yields than that on spin-coating thin films. According to the temperature-dependent spectra, the conjugated polygrid PODPFG with lower quantum efficiencies on micrometer-scale thick films exhibits the J-aggregate emission, while the micrometer-thick multilayer crack films of PG-Cz exhibit the H-aggregate emission (also including the PG-Cz-based microsphere films with similar H-aggregate emission, in Figure S46). Thus, the enhancement of quantum yields is more related to the H-aggregate emission of PG-Cz interchain behaviors. Moreover, according to the excitation spectra in Figure S41-43 in the Supplementary Materials, the thin film with low quantum yields merely has an excitation peak on the single-chains (at 360 nm, corresponding to the excitation of tetrafluorene moieties), while the micrometer-thick films with higher quantum yields have long-wavelength excitation peaks in the ranges of 380~410 nm (assigned to the excitation from the interchain aggregation).

For the contribution from single-chain conformation, the planar conformation of polyfluorene single-chains is favorable to enhance the quantum yields in the aggregate state, according to our previous works about crystallization-induced emission enhancement, where the conformational planarization of polyfluorenes derivatives supports the crystallization behaviors and the increase in quantum yields. However, the Raman spectra reveal that the polygrid PG-Cz displays the torsional main-chains (Figure S5) rather than the planarized conformation. What’s more, the conformation modes are almost the same among spin-coating thin films, multilayer crack films, and microsphere films. Thus, the contribution of single-chain conformation to the enhancement of quantum yields is ruled out.

**Demonstrations on random lasing**


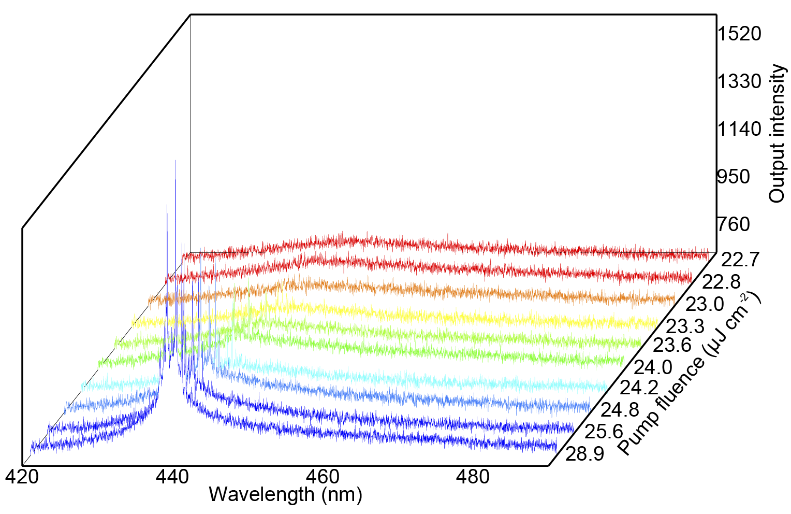


**Figure S48: The changes of random lasing intensity as the variation of energy density (22.7~28.9 μJ cm^-2^) on PG-Cz-based drop-casting film with massive material/air interfaces.**


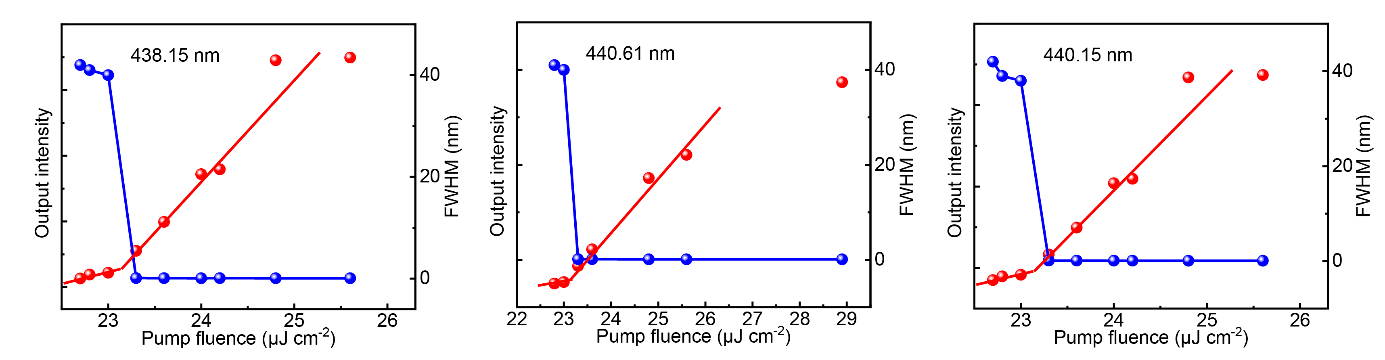


**Figure S49: The threshold calculation on other lasing emission peaks at 438.15 nm, 440.15 nm, and 440.61 nm.**


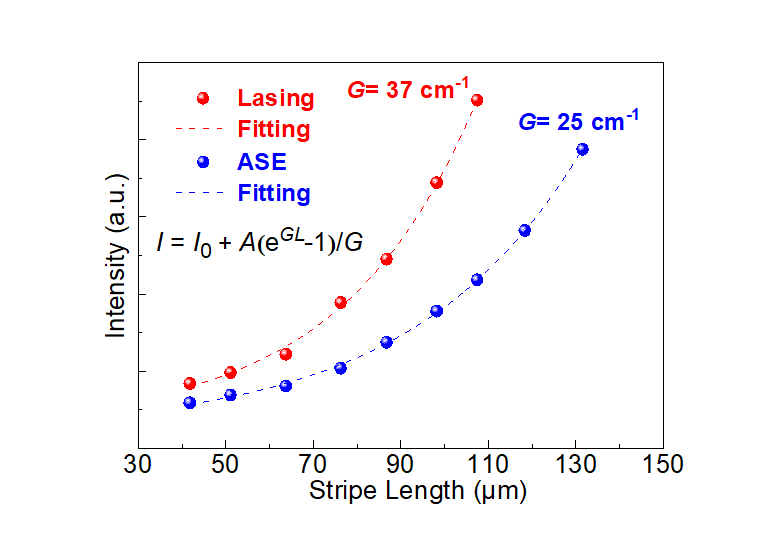


**Figure S50: The gain coefficient of ASE (from the solvent-annealing film) and lasing (from the drop-casting film).**

**Figure S51: The statistic calculation of quality factors among various lasing peaks.**

**Figure S52: Magnifying the lasing spectra in a range of 1 nm width.**


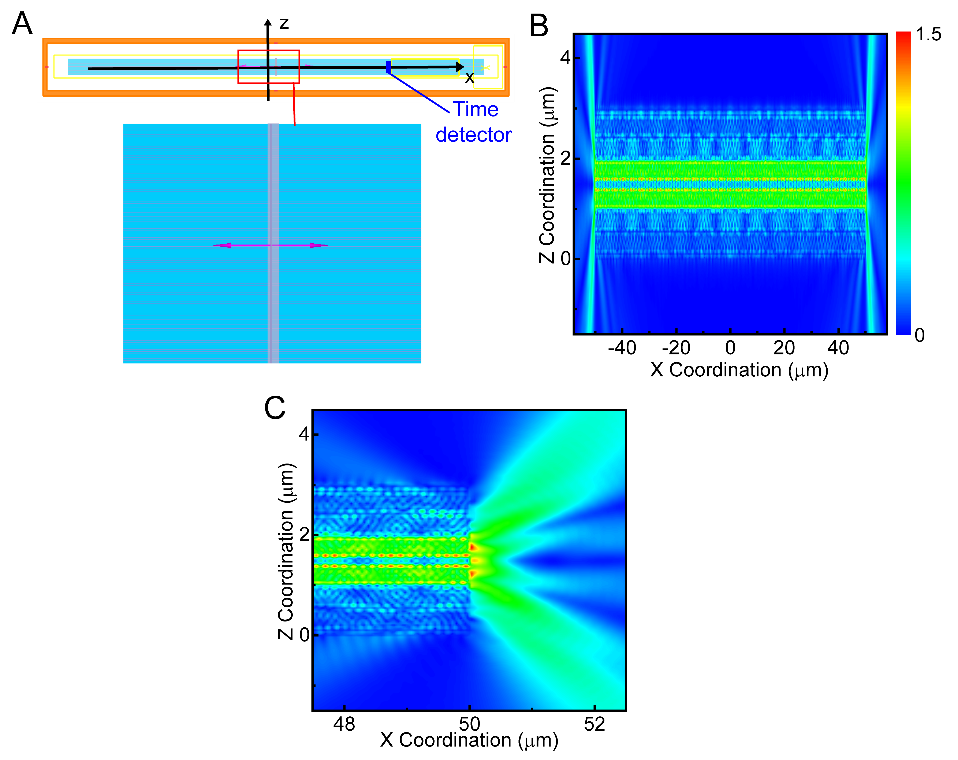


**Figure S53: The FDTD simulation on multilayer stacking films (∆*n* = 1.1).** The distribution of electric field intensity mapping is shown in (B), where (C) shows the magnitude picture at the edge.


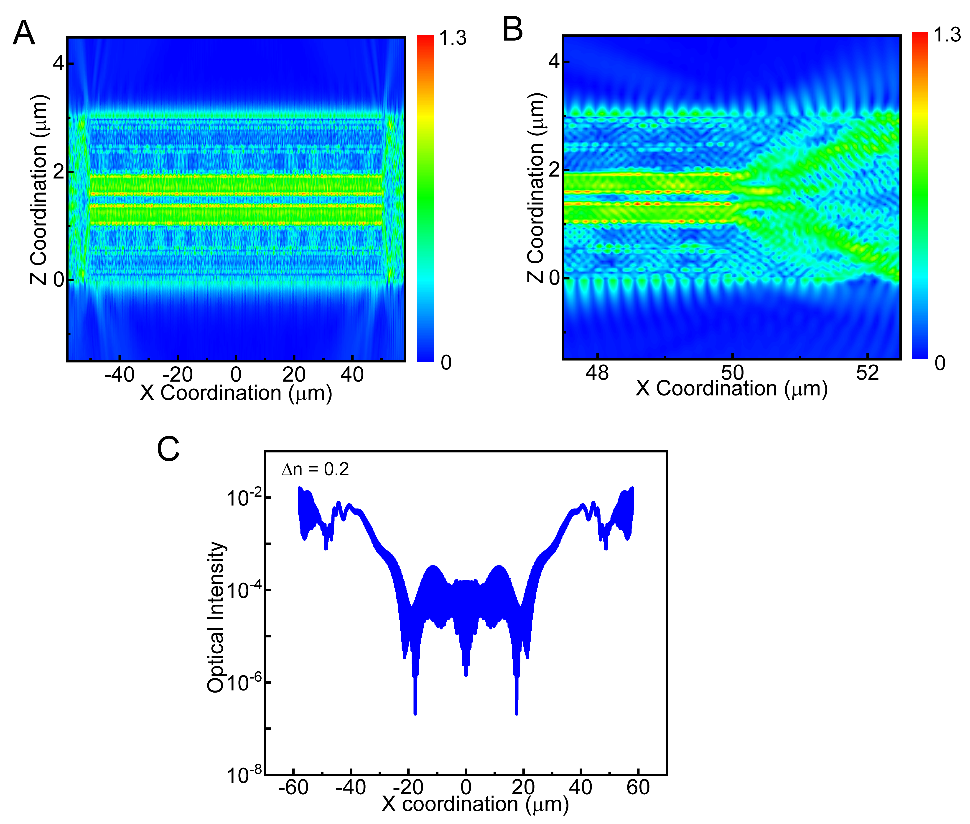


**Figure S54: The FDTD simulation on multilayer stacking films with ∆*n* = 0.2.** The distribution of electric field intensity mapping is shown in (B), where (C) shows the magnitude picture at the edge.

**
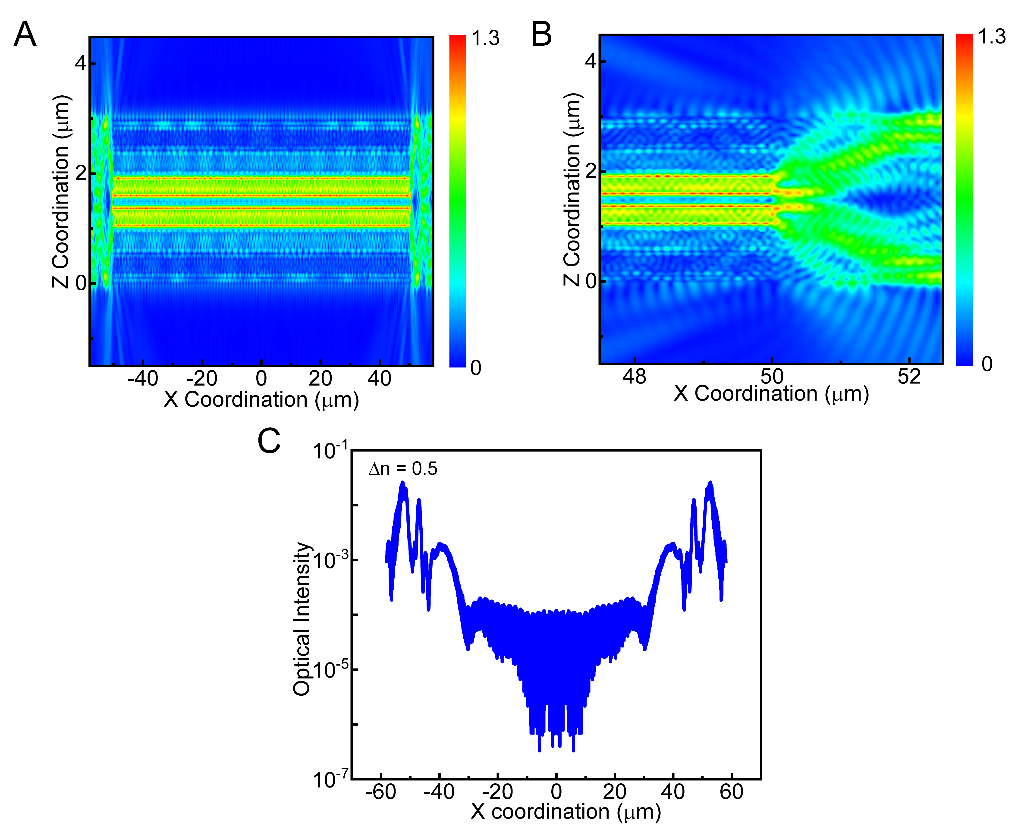
**

**Figure S55: The FDTD simulation on multilayer stacking films with ∆*n* = 0.5.** The distribution of electric field intensity mapping is shown in (B), where (C) shows the magnitude picture at the edge.


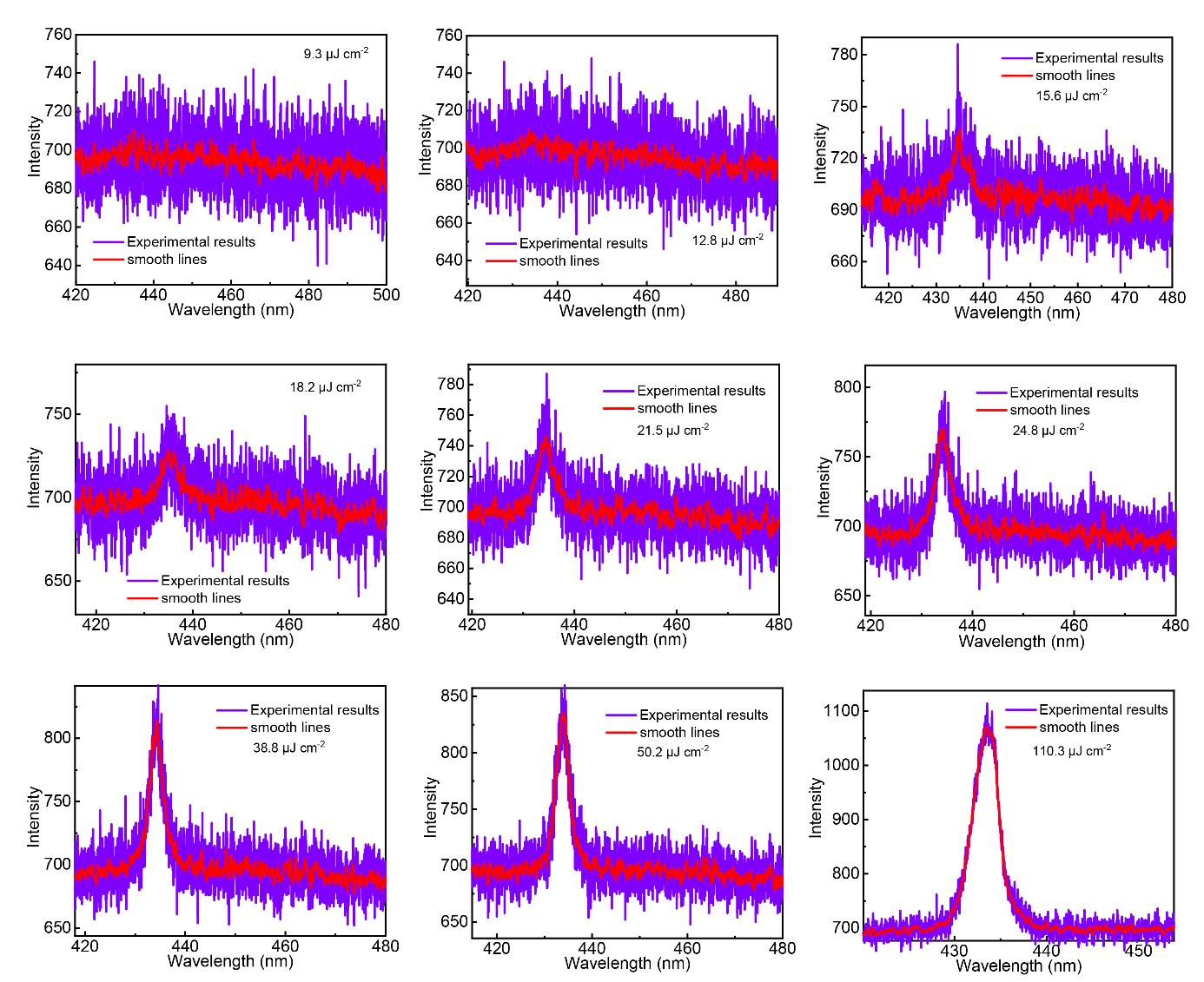


**Figure S56: The changes of ASE intensity as the variation of energy density (18~50 μJ cm^-2^) on PG-Cz-based solvent-annealing film with the attachment of PMMA-based solvent-casting film.** As the resolution is poor under low energy density, we smooth these emission spectra lines to tentatively analyze their intensity and linewidth. We observed the relatively narrow emission peaks (like ASE) at 435 nm under 15~18 μJ cm^-2^, which can be near the ASE threshold. The PMMA film is used to cover the PG-Cz layer to diminish interface crack and enhance ASE stability for the threshold calculation.


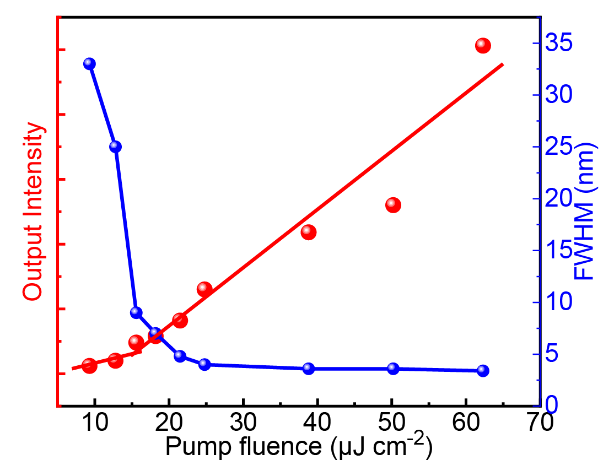


**Figure S57: The threshold calculation of ASE action on PG-Cz-based solvent-annealing film with the attachment of PMMA-based solvent-casting film.** The threshold is evaluated as ~16 μJ cm^-2^. The PMMA film is used to cover the PG-Cz layer to diminish interface crack and enhance ASE stability for the threshold calculation.


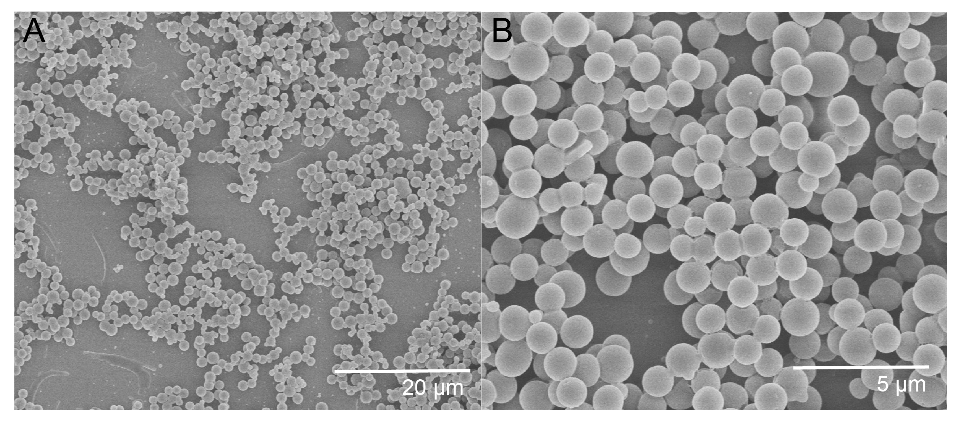


**Figure S58: The SEM image of PG-Cz-based microsphere film.**


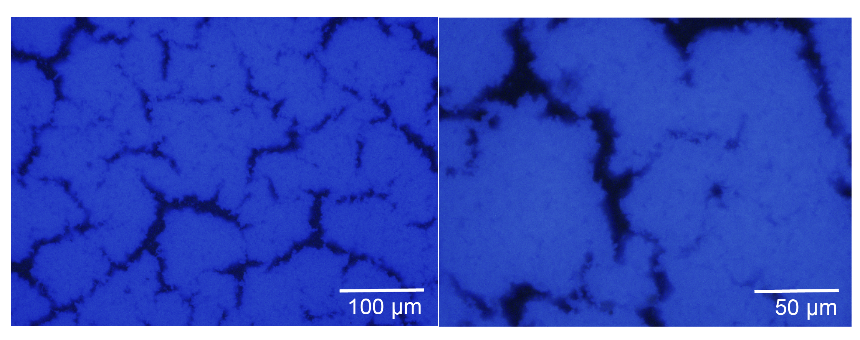


**Figure S59: The fluorescence microscopy image of PG-Cz-based microsphere film.**

**
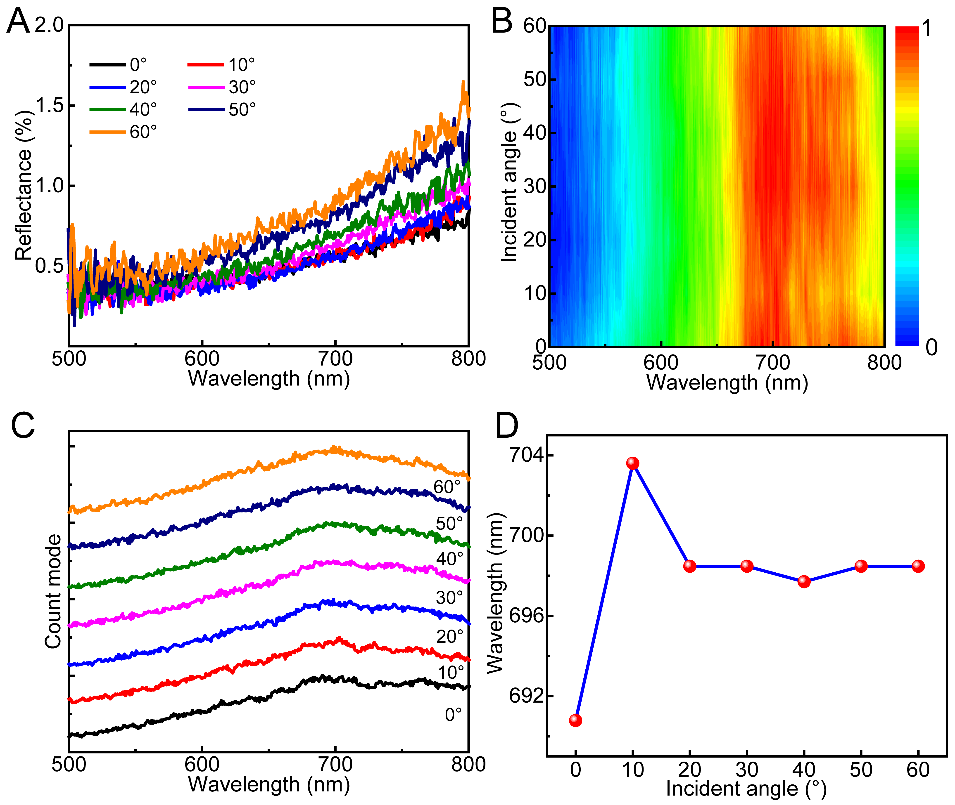
**

**Figure S60: The angle-dependent reflectance spectra of PG-Cz-based microsphere film.** (A) The wavelength-dependent absolute reflectance in the range of 500~800 nm wavelength and 0~60° incident angle. (B) The two-dimensional count mode mapping. (C) The one-dimensional count mode in the incident angle ranges of 0~60°. (D) The relationship between the wavelength peak and the incident angle. These results with broad peaks and angle-independent reflectance reveal that there is no photonic crystal feature on microsphere film. The extremely low absolute reflectance is derived from the serious random light scattering on rough film surfaces.


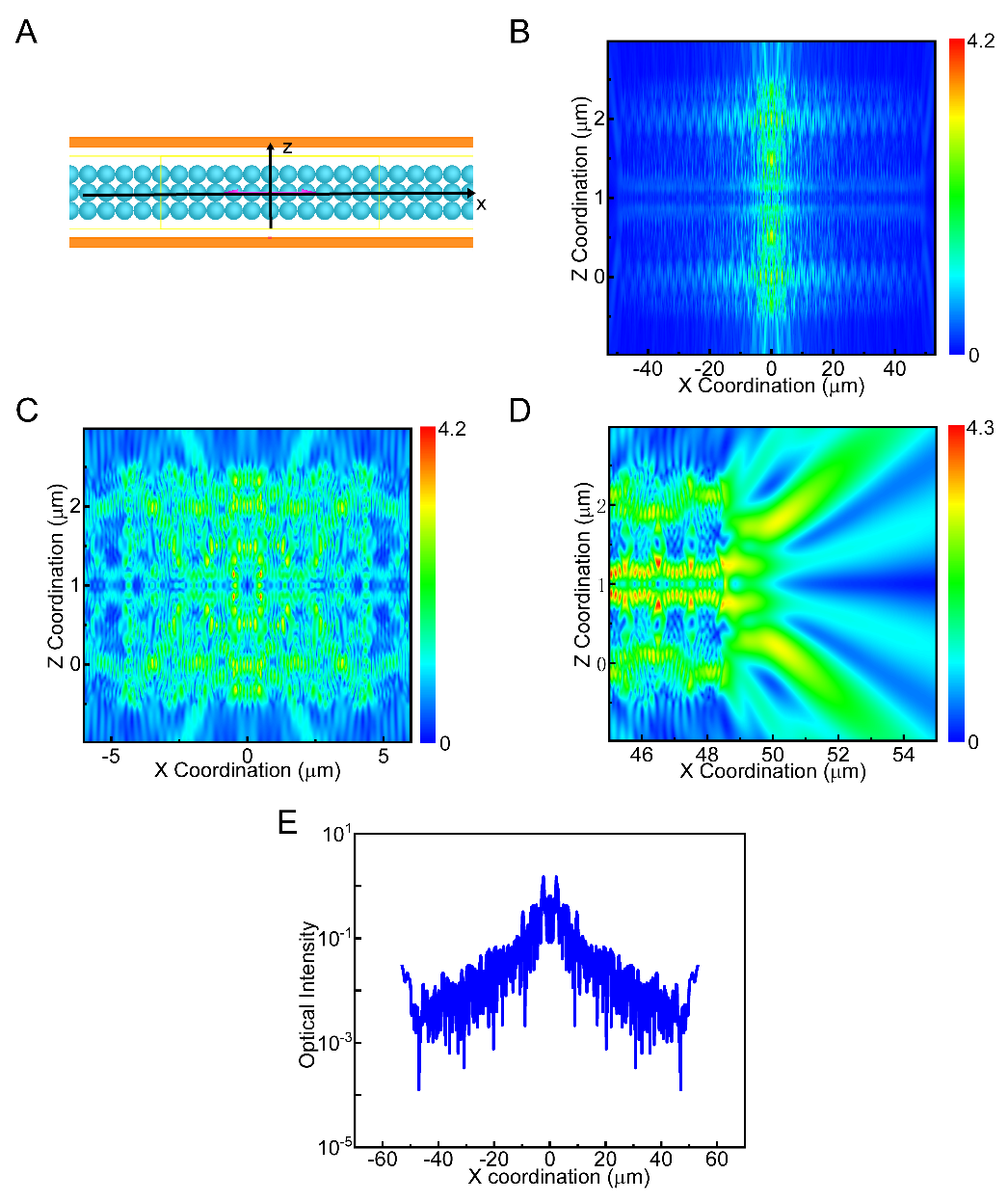


**Figure S61: The FDTD simulation on microsphere films.** The distribution of electric field intensity mapping is shown in (B) and (C).

**Reference**

[1] B. Liu, J. Lin, F. Liu, M. Yu, X. Zhang, R. Xia, T. Yang, Y. Fang, L. Xie, W. Huang, “A Highly Crystalline and Wide-Bandgap Polydiarylfluorene with β-Phase Conformation toward Stable Electroluminescence and Dual Amplified Spontaneous Emission,” *ACS Applied Materials & Interfaces* vol. **8**, pp. 21648-21655, 2016.

[2] D. Lin, Y. Wei, A. Peng, H. Zhang, C. Zhong, D. Lu, H. Zhang, X. Zheng, L. Yang, Q. Feng, L. Xie, W. Huang, “Stereoselective gridization and polygridization with centrosymmetric molecular packing,” *Nature Communications* vol. **11**, pp. 1756-1766, 2020.

[3] Y. Wu, J. Xu, E. T. Poh, L. Liang, H. Liu, J. K. W. Yang, C.-W. Qiu, R. A. L. Vallée, X. Liu, "Upconversion superburst with sub-2 μs lifetime," Nature Nanotechnology vol. 14, no. 12, pp. 1110-1115, 2019.

[4] M. Pelton, "Modified spontaneous emission in nanophotonic structures," Nature Photonics vol. 9, no. 7, pp. 427-435, 2015.
